# Atmospheric oxidation capacity at northern mid-latitude regions is reaching a turning point

**DOI:** 10.1093/nsr/nwag178

**Published:** 2026-03-24

**Authors:** Liyan Wei, Shaw Chen Liu, Zhaofeng Tan, Xuefei Ma, Ming Zhou, Wenyu Wei, Jingwen Zhang, Yuanhang Zhang, Run Liu, Keding Lu

**Affiliations:** State Key Laboratory of Regional Environment and Sustainability, College of Environment Sciences and Engineering, Peking University, Beijing 100871, China; Institute for Environmental and Climate Research, College of Environment and Climate, Jinan University, Guangzhou 511443, China; State Key Laboratory of Regional Environment and Sustainability, College of Environment Sciences and Engineering, Peking University, Beijing 100871, China; State Key Laboratory of Regional Environment and Sustainability, College of Environment Sciences and Engineering, Peking University, Beijing 100871, China; State Key Laboratory of Regional Environment and Sustainability, College of Environment Sciences and Engineering, Peking University, Beijing 100871, China; State Key Laboratory of Regional Environment and Sustainability, College of Environment Sciences and Engineering, Peking University, Beijing 100871, China; Institute for Environmental and Climate Research, College of Environment and Climate, Jinan University, Guangzhou 511443, China; State Key Laboratory of Regional Environment and Sustainability, College of Environment Sciences and Engineering, Peking University, Beijing 100871, China; Institute for Environmental and Climate Research, College of Environment and Climate, Jinan University, Guangzhou 511443, China; State Key Laboratory of Regional Environment and Sustainability, College of Environment Sciences and Engineering, Peking University, Beijing 100871, China

**Keywords:** hydroxyl radical, observation-based method, atmospheric oxidation capacity, turning point

## Abstract

The hydroxyl radical (OH) constitutes the primary atmospheric oxidant, governing the removal of short-lived greenhouse gases such as methane and the formation of secondary pollutants, including ozone. Characterizing long-term OH variability is critical for understanding atmospheric oxidation capacity and formulation of air quality improvement strategies. Although previous studies have linked OH increases to anthropogenic emissions, particularly nitrogen oxides (NO_x_), the historical evolution of OH remains poorly constrained. Here, we develop an observation-based method (OBM) to quantify spatiotemporal variability of surface OH concentration and identify its key turning points for China, Europe and the United States. Relative to clean regions, OH concentrations have increased by about 300% in all three regions, mainly attributable to enhanced NO_x_ emissions. However, OBM-derived trends reveal that OH levels have reached a turning point, with statistically significant decreases observed at ∼10% of stations in Europe and the United States, while ∼85% of stations continue to increase. At hemispheric mid-latitudes, OH levels have remained near peak values of (5–9) × 10^6^ cm^−3^ over the past 50–60 years, and it may take another several decades to fully return to the NO_x_-limited regime, beyond the turning point. These findings challenge prior assessments of OH increases or stability and suggest an emerging decline in mid-latitude atmospheric oxidation capacity, as well as provide a novel indicator of regional ozone control strategies.

## Teaser

Under continued NO_x_ mitigation, atmospheric boundary layer OH concentrations in the USA and Europe are found to have reached a turning point, moving from NO_x_-saturated conditions to NO_x_-limiting conditions, while the OH concentrations in China are still in NO_x_-saturated conditions. These uncovered OH trends can have profound implications for regional ozone control strategies as well as the global methane lifetime.

## INTRODUCTION

Trace gases such as nitrogen oxides (NO_x_), methane (CH_4_), carbon monoxide (CO), and volatile organic compounds (VOCs) exert a substantial impact on the chemical composition and radiative properties of the atmosphere [1–3]. The hydroxyl radical (OH), as the dominant atmospheric oxidant, governs the removal of these trace gases and mediates the formation of secondary pollutants, thereby playing a pivotal role in both air quality and climate change [4–6]. The atmospheric oxidation capacity by OH is primarily generated via the photolysis of ozone (O_3_), followed by reactions of the resulting excited oxygen atoms with water vapor [7,8]. Its sustained presence is maintained through chain-propagating reactions involving VOCs, which produce hydrogen peroxy radicals (HO_2_ and RO_2_) that regenerate OH via reactions with nitric oxide (NO). Removal pathways of OH differ depending on the local NO_x_ regime [9]: under high-NO_x_ conditions, OH is mainly lost through reaction with NO_2_, forming nitric acid (HNO_3_) or nitrous acid (HONO), while under low-NO_x_ condition, radical-radical self-reactions dominate, leading to the forming of hydrogen peroxide (H_2_O_2_) or organic hydroperoxides (ROOH). The dependence of OH concentration on NO_2_ exhibits a nonlinear structure: at low NO_x_ levels, OH increases with NO_2_ due to enhanced recycling via HO_2_ + NO, while at high NO_x_ levels, OH decreases owing to increased termination through HNO_3_ formation [8]. The maximum OH concentration typically occurs at intermediate NO_2_ concentrations of a few parts per billion (ppb), outlining a turning point that separates NO_x_-limited and NO_x_-saturated regimes.

Our previous study conducted in Guangdong Province characterized the nonlinear response of surface OH concentrations to ambient NO_2_ levels (Fig. 6 in [10]). The analysis indicated that OH concentrations increased substantially under sustained NO_x_ emissions since preindustrial times, as NO_2_ remained within the range of 0.001–3 ppb. OH peaked at approximately 3 ppb NO_2_, consistent with the theoretical turning point, beyond which further increases in NO_2_ led to suppression of OH due to enhanced radical termination. In this high-NO_x_ regime, subsequent reductions in NO_x_ emissions during 2015–2020 were associated with rising OH levels, reflecting a reversal in the oxidative response. However, this prior work was restricted to urban environments and lacked spatial coverage beyond the regional scale. As a result, larger regional or even hemispheric distribution of OH sensitivity to NO_2_ remains poorly resolved.

Long-term, spatially resolved observations of OH concentration remain limited. At the hemispheric or global scale, OH levels are primarily inferred from atmospheric chemistry models and halocarbon-based inverse modeling. Simulations from the Coupled Model Intercomparison Project Phase 6 (CMIP6) indicate an approximate 9% increase in tropospheric OH since 1980 [11–13], whereas other studies report negligible trends over recent decades [14–16]. At regional scales, box models and chemical transport models applied in urban Beijing and the Beijing–Tianjin–Hebei region have reported increasing OH levels and high spatial heterogeneity, largely attributed to anthropogenic emissions [17,18]. However, substantial inter-model discrepancies persist, with uncertainties reaching up to 50%, primarily due to limitations in emission inventories and the presentation of chemical mechanism [19–22]. Although global tropospheric ozone (a key OH source) has increased by approximately 40% since 1850 due to anthropogenic activity [13], the corresponding OH response, particularly in the northern hemisphere, remains poorly constrained. To address this gap, an observation-based method (OBM) is applied [23], employing photochemical age diagnostics under Lagrangian assumptions in conjunction with box model simulations and historical observational data, to quantify long-term trends and identify NO_x_-included turning points in surface OH concentrations across hemispheric mid-latitudes.

## RESULTS AND DISCUSSION

### Distributions and trends of OH concentrations

**Figure 1.**
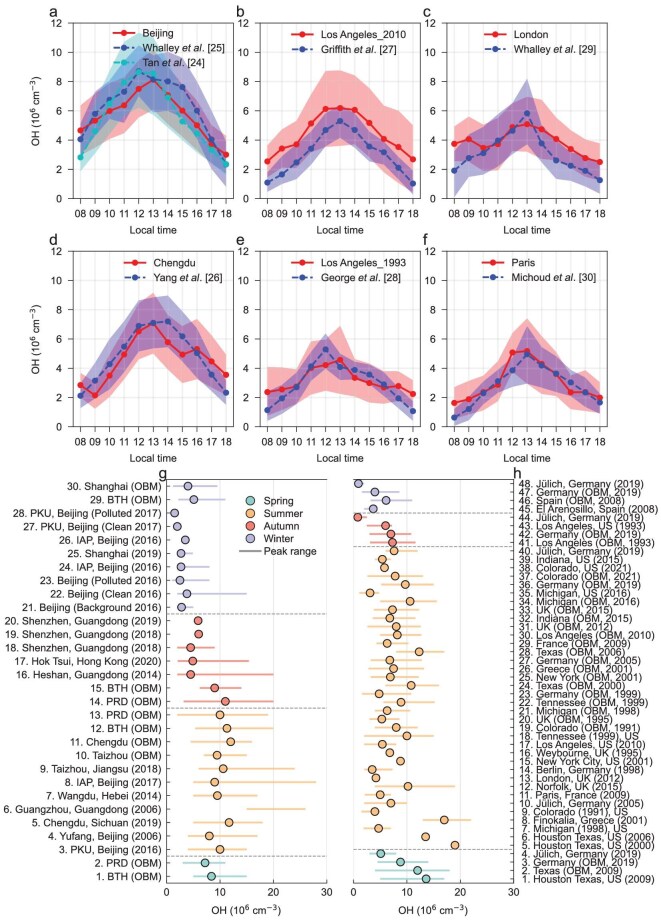
Comparison of hourly OH concentrations. OH values (08:00–18:00 LT) calculated by OBM in Beijing (Jun–Aug 2017) (a), Los Angeles (May–Jun 2010; September 1993) (b, e), London (Jun–Aug 2012) (c), Chengdu (Aug–Sep 2019) (d), and Paris (July 2009) (f) (solid line with shade) are compared with those OH observations in Beijing [24,25] (blue dashed line with blue shade; cyan dashed line with cyan shade), Los Angeles [27,28], London [29], Chengdu, and Paris [26,30] (blue dashed line with blue shade). Shaded areas represent the range of mean values ± the 0.5 standard deviation for each city. Comparison between average OH peak values from previously reported measurements and OBM method over China (g), the United States, and Europe (h) at different seasons and time periods. (g): No. 3 adapted from ref [52]. No. 4 adapted from ref [53]. No. 5 adapted from ref [26]. No. 6 adapted from ref [54]. No. 7 adapted from ref [24]. No. 8 adapted from ref [25]. No. 9 adapted from ref [47]. No. 16 adapted from ref [55]. No. 17 and No. 18 adapted from ref [26,56]. No. 19 adapted from ref [26]. No. 20 adapted from ref [57]. No. 21, No. 22, and No. 23 adapted from ref [58]. No. 24 adapted from ref [59]. No. 25 adapted from ref [60]. No. 26 adapted from ref [59]. No. 27 and No. 28 adapted from ref [61]. (h): No. 1 adapted from ref [62]. No. 4 adapted from ref [36]. No. 5 and No. 6 adapted from ref [34]. No. 7 adapted from ref [63]. No. 8 adapted from ref [64]. No. 9 adapted from ref [65]. No. 10 adapted from ref [66]. No. 11 adapted from ref [30]. No. 12 adapted from ref [67]. No. 13 adapted from ref [29]. No. 14 adapted from ref [68]. No. 15 adapted from ref [34]. No. 16 adapted from ref [69]. No. 17 adapted from ref [27]. No. 18 adapted from ref [70]. No. 35 adapted from ref [71]. No. 38 adapted from ref [9]. No. 39 adapted from ref [72]. No. 40 adapted from ref [36]. No. 43 adapted from ref [28]. No. 44 adapted from ref [36]. No. 45 adapted from ref [73]. No. 48 adapted from ref [36].

To quantify the spatial and temporal distribution of surface OH concentrations from 1985 to 2023, an observation-based method was applied using ground-based measurements of NO_2_, CO observations across China, the United States, and Europe (see OH calculation in Methods). The OBM-derived OH concentrations exhibit strong consistency with previously reported measurements (Fig. 1). In China, mean OBM OH concentrations, including a ± 0.5 standard deviation, deviate by 4%–25% from summer observations recorded in Wangdu (2014) and urban Beijing (2017), while differing by −31% to 34% from measurements taken in Chengdu during Aug–Sep 2019 [24–26]. In Los Angeles, OBM values differ by −20% to 51% compared to filed campaigns conducted in May–Jun 2010 and September 1993 [27,28], however, the overlapping uncertainty bounds suggest reasonable agreement. Similarly, OBM OH concentrations show deviations of 5%–47% above July 2012 observations in London under fine weather conditions [29] and −22% to 32% below July 2009 measurements in Paris [30]. Additionally, we conducted comparisons with results by empirical parametrization methods in the Beijing–Tianjin–Hebei region [31,32] (Fig. S2), simulations from regional air quality model in Guangdong Province (Fig. 6 in [10]), and observational data from seasonal and temporal patterns across three regions (Fig. 1g–h), along with corresponding tests (Fig. 4). These results also demonstrated strong consistency between OBM-derived OH levels, field measurements, and model simulations. Moreover, in the United States with low NO_x_ (∼1 ppb), our OBM method remains effective, yielding relatively low OH values on the left regime of the turning point on the dependence curve of OH on NO_2_. This aligns with the theoretical expectation that extremely low NO_x_ concentrations correspond to lower OH levels. Meanwhile, under high-NO_x_ environment (∼10 ppb), larger OBM OH values distribute around and to the right regime of the turning point, which is also consistent with the generally higher simulated and observed OH values currently reported [8]. Collectively, these comparisons support the robustness and reliability of the OBM OH estimation framework applied in this work.

**Figure 2.**
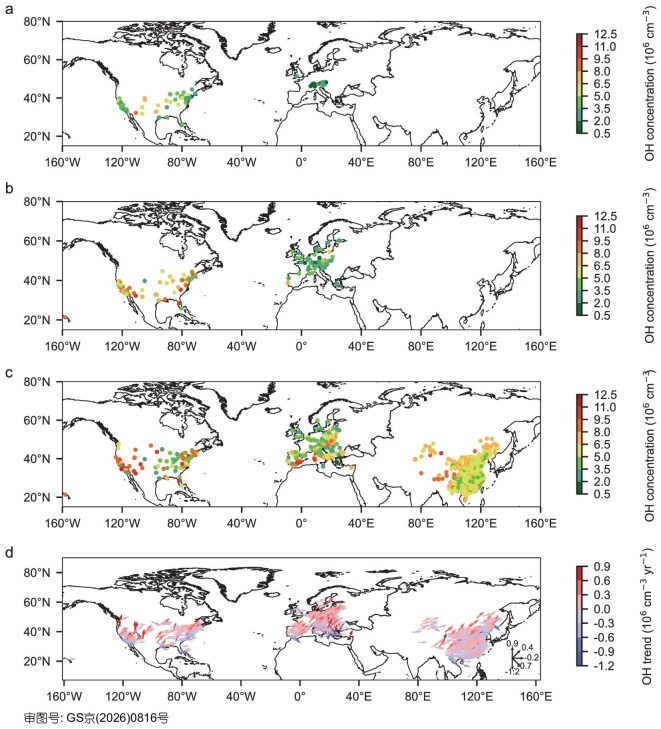
Spatial distribution patterns. OH daytime (08:00–18:00 LT) concentrations over 1985–1999 (a), 2000–2014 (b), and 2015–2023 (c), and annual increases over 2015–2023 (d) in China, the United States, and Europe, respectively. An arrow pointing at 90° (vertically upward) corresponds to the maximum positive increase, while an arrow pointing at −90° (vertically downward) indicates the maximum negative increase. The continuous variation of angles within this range represents the transition of the annual increase from positive to negative.

Substantial temporal and spatial variability in daytime OH levels (08:00–18:00 LT) are evident over the period 1985–2023 (Fig. 2a–c). Between 1985 and 1999, the mean daytime OH concentration in the United States was (4.1 ± 1.5) ×10^6^ cm^−3^, approximately 71% higher than in Europe ((2.4 ± 1.0) ×10^6^ cm^−3^). During 2000–2014, OH concentrations in the United States increased to (6.1 ± 2.1) ×10^6^ cm^−3^, remaining a ∼79% enhancement over Europe ((3.4 ± 1.4) ×10^6^ cm^−3^). By 2015–2023, OH levels in the United States reached (6.5 ± 2.5) ×10^6^ cm^−3^, remaining about 10% and 27% higher than those in China ((5.9 ± 1.1) ×10^6^ cm^−3^) and Europe ((5.1 ± 2.0) ×10^6^ cm^−3^). Spatially, higher OH concentrations are observed in western and northern China, particularly across the Tibetan Plateau, where intense solar radiation enhances photochemical activity. Similarly, high OH levels occur in the western United States and southern Europe, driven by high O_3_ concentrations and precursor emissions that promote HO_2_ propagation and subsequent OH production [33].

Between 2015 and 2023, the most rapid increases in surface OH concentrations were observed in Europe, with a mean annual increase of 10.2 × 10^4^ cm^−3^ and statistically significant positive trends (*p <* 0.05) identified at ∼41% of stations (Figs 2d and S3). In comparison, the United States and China exhibited lower annual increases of 9.4 × 10^4^ and 6.5 × 10^4^ cm^−3^, respectively, with significant trends observed at ∼33% of stations in both regions. The regional heterogeneity in OH trends appear to reflect differences in the magnitude of annual NO_2_ decreases. Europe experienced the steepest decline in NO_2_ concentrations (0.5 ppb), likely leading to less OH loss through the OH + NO_2_ pathway. In contrast, smaller NO_2_ decreases were recorded in China (0.4 ppb) and the United States (0.3 ppb). Spatially, the strongest OH increases were concentrated in western and northern China, the central and eastern United States, and northern Europe. These areas with high OH growth dominate the positive OH trends over China, United States, and Europe.

**Figure 3.**
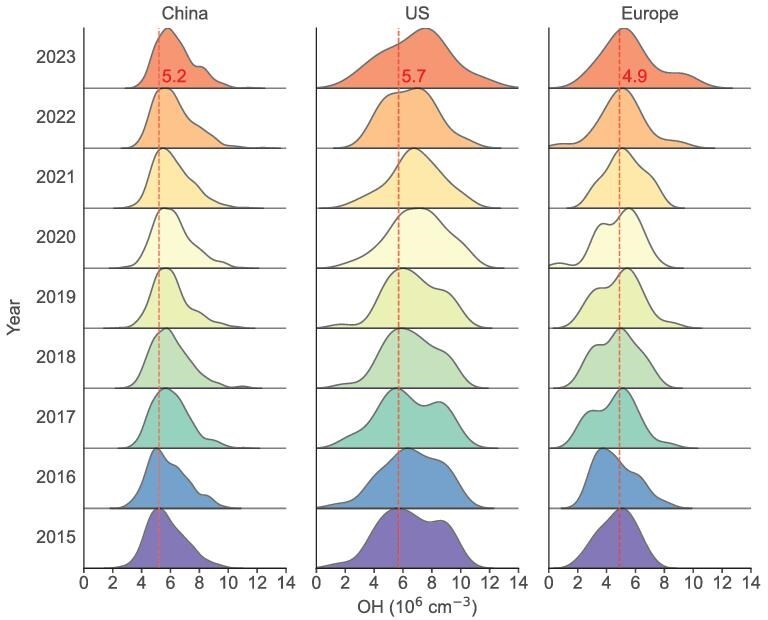
Distributions of annual mean OH in China (*N* = 331), the United States (*N* = 37) and Europe (*N* = 21) over 2015–2023.

Strong positive trends in surface OH concentrations have been identified in both the United States and Europe over the past three decades (Fig. S4). A reanalysis of long-term data from 53 stations in the United States, each with a minimum of 25 years of observations, and 26 stations in Europe with at least 22 years of data, reveals statistically significant increases in OH. OH concentrations exhibit a significant upward trend from 1991 to 2021 (*R* = 0.97, *p <* 0.01) in the United States, while a similar trend is observed from 1995 to 2022 (*R* = 0.92, *p <* 0.01) in Europe. Looking across more areas, we also find that in the past decade, OH concentrations in all three regions have shown an increasing trend (Fig. 3). Compared to 2015, more OH values in 2023 fall to the right of the red dashed line, indicating a growing proportion of high OH levels.

The results presented above reveal robust positive trends in annual surface OH concentrations across China, the United States, and Europe. These findings are broadly consistent with previous estimates derived from numerical modeling. For instance, Wang *et al.* reported a near doubling of ground-level OH concentrations in urban Beijing between 2006 and 2020, based on a constrained box model [18]. Similarly, multi-model simulations from global chemistry-climate ensembles have indicated persistent increases in mid-latitude tropospheric OH since 1980 [11,12].

In contrast to the increasing trend in OH concentrations, anthropogenic emissions in all three regions have declined to varying degrees due to air pollution control measures. To classify anthropogenic emissions across different regions and time scales, a key approach is to analyze long-term trends in emission inventories, differentiating them by their policy-driven transition periods specific to each region. Meanwhile, by modifying the abundances of multiple trace gases, anthropogenic emissions exert substantial influences on the production, loss, and recycling of atmospheric OH. It is therefore necessary to quantify the impacts of anthropogenic emissions on OH across different regions.

### The impact of anthropogenic emissions on OH concentrations

**Figure 4.**
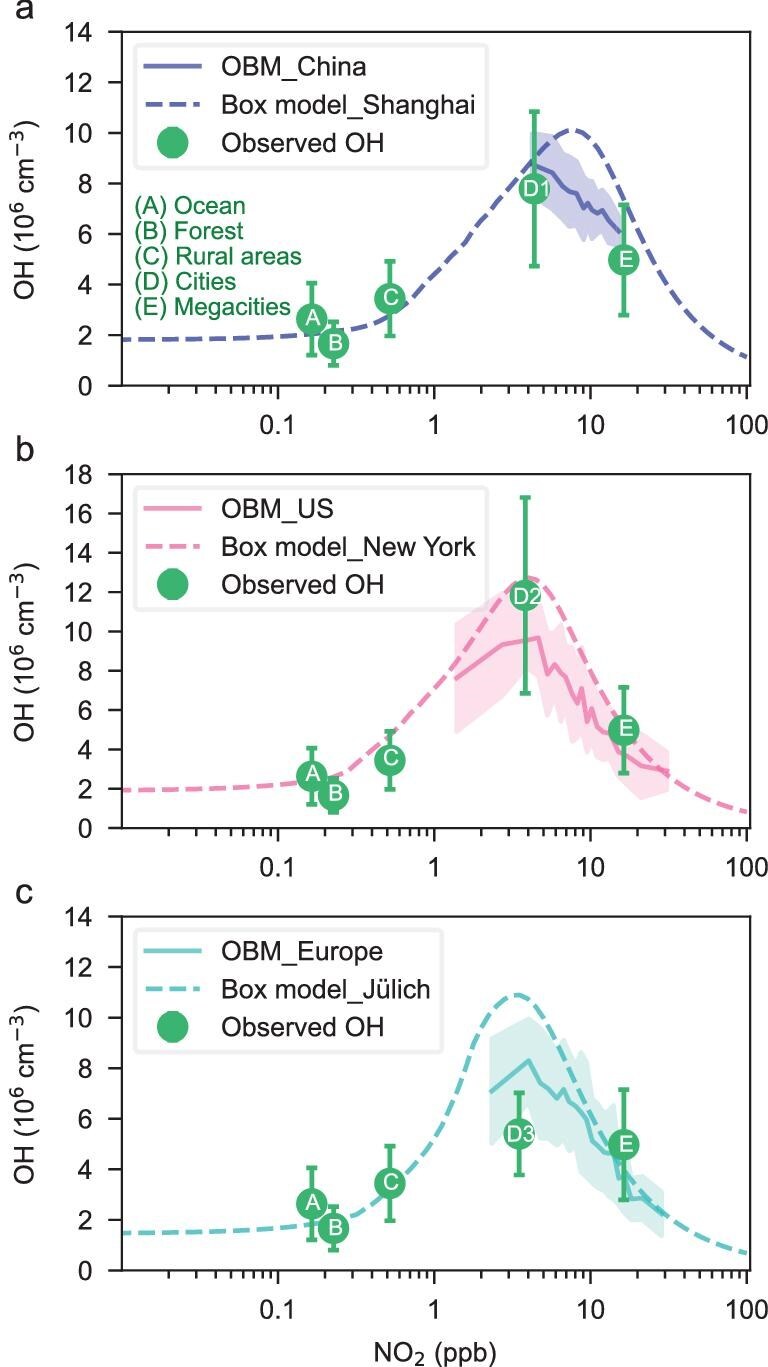
The dependence of OH on NO_2_. Noontime (11:00–13:00 LT) OH concentrations derived by the OBM during 2015–2023 for China (*N* = 331) (a, solid line with 1-sigma shade), 1985–2023 for the United States (*N* = 535) (b, solid line with 1-sigma shade), and Europe (*N* = 594) (c, solid line with 1-sigma shade), are compared with simulations by the chemical box model for China (Shanghai, dashed line), the United States (New York, dashed line), Germany (Jülich, dashed line), respectively, and observations (green dots with one standard deviation vertical bars). Points D1, D2, D3 represent measurements in Chinese cities, US cities and European cities, respectively. Shaded areas represent the range of mean values ± 1 standard deviation for each region.

Noontime OH concentrations estimated by the OBM during 2015–2023 for China, 1985–2023 for the United States and Europe are presented by blue, pink, and cyan solid curves with shade areas, respectively (Fig. 4). These OBM values are compared against OH simulations derived from chemical box models for representative stations (Table S1): Shanghai (blue dashed line), New York [34,35] (pink dashed line), and Jülich [36] (cyan dashed line) and observations (green dots with one standard deviation vertical bars). In this study, according to geographic region and NO_2_ concentration level, OH observations from field campaigns are classified into five categories, i.e., forest (areas with abundant vegetation and minimal anthropogenic emissions, NO_2_: 0–0.5 ppb), ocean (open seas and remote islands, NO_2_: 0–1 ppb), rural area (areas far from cities, near forest edges, and coastal bays, NO_2_: 0–1 ppb), city (towns, suburbs of large cities, and coastal bays, NO_2_: 1–10 ppb) and megacity (metropolitan areas or large economic zones, NO_2_: ≥10 ppb) (Table S5). The chemical box model for OH calculations on a broad range of NO_2_ is described in Methods section (Chemical box model and Model setup for the preindustrial scenario). NO_2_ concentrations from corresponding OBM-derived OH range from 4 to 15 ppb in China, 1 to 30 ppb in the United States, and 2 to 29 ppb in Europe, suggesting moderately polluted conditions in China, and a spectrum from relatively clean to polluted conditions across the United States and Europe. NO_2_ concentrations have increased from extremely low levels (0.1–0.3 ppb) during the preindustrial period to 3 ppb due to increased anthropogenic emissions caused by the industrial revolution, subsequently entering regime NO_2_ over 3 ppb. However, in recent three decades, due to the implementation of stringent pollution control measures, especially since 1985, there has been a noticeable downward trend in anthropogenic NO_x_ emissions in the United States and Europe, and similarly in China since 2015 (Fig. S6a). The NO_2_ concentration ranges corresponding to the OBM OH values also show a significant downward trend and remains below 3 ppb at some monitoring stations, particularly in the United States, highlighting its most effective emission reduction efforts.

OBM-derived OH concentrations in China exhibit good agreement with measurements from city and megacity environments (green dots labeled D1 and E in Fig. 4) and are consistent with chemical box model simulations for Shanghai. In the United States, OBM OH levels correspond well with observations in cities and megacities (green dots labeled D2 and E) but notably lower than box model outputs from New York. In Europe, OBM OH values are higher than observations in cities (green dot labeled D3), but slightly lower than box model estimates for Forschungszentrum Jülich (FZJ), with relative differences of less than 30%. Notably, box model simulations tend to produce higher OH values or peak at higher NO_2_ levels compared to OBM results. These discrepancies likely stem from temporal mismatches, as box model simulations are typically constrained to single-season conditions (e.g., summer or autumn), while OBM OH and observational datasets reflect multi-seasonal averages. Under same season conditions, OBM OH concentrations from three regions align more closely with the simulated results from the box model (Fig. S7). Therefore, these comparisons suggest that OBM-derived OH concentrations are broadly consistent with both direct observations and box model simulations, while offering seasonally integrated insight into regional oxidation capacity.

The box model results from three regions show that the simulated OH concentrations under low-NO_x_ environments are consistent with those observed in the relatively clean regions of the ocean and forest (green dots labeled A and B in Fig. 4). As anthropogenic emissions increase, the rural results (green dot labeled C) begin to show the increase in OH as NO_2_ increases. This is clearly illustrated in Fig. 4, with the agreement of the box model with the measurements for these areas. In China, both observational data and chemical box model simulations indicate that OH concentrations under polluted conditions are approximately 3 to 5 times higher than those observed in pristine atmospheres with NO_2_ levels ≤ 0.3 ppb, indicating that the dominant influence of anthropogenic emissions on atmospheric oxidation capacity. The mean OH concentration in China, as represented by the OBM-derived estimate (blue curve), is 7.3 × 10^6^ cm^−3^, compared to a pristine baseline of 2.1 × 10^6^ cm^−3^ (Forest and Ocean: green dots at NO_2_  ≤ 0.3 ppb), corresponding to an anthropogenic enhancement factor of 3.5. The mean OH values in the United States (pink curve) and Europe (cyan curve) are 6.4 × 10^6^ and 5.4 × 10^6^ cm^−3^, relative to a pristine estimate of 2.1 × 10^6^ cm^−3^, yielding enhancement factors of 3.0 and 2.6, respectively. These results demonstrate a consistent and substantial anthropogenic impact on present-day surface OH concentrations across all three regions, relative to clean regions.

The OH budget exhibits substantial differences between present-day and pristine scenarios (Fig. S5), reflecting shifts in chemical regimes driven by anthropogenic emissions. Under present-day scenarios, noontime (11:00–13:00 LT) OH production in Shanghai, New York, and Jülich is mainly dominated by the radical recycling reaction HO_2_ + NO, contributing 73%–76%. The primary sources include photolysis of HONO (HONO + *hv*), accounting for 8%–15%, and O_3_ photolysis followed by the O(^1^D) + H_2_O reaction pathway, contributing approximately 6%. OH losses in Shanghai, New York, and Jülich, are primarily associated with reactions involving OVOCs (28%–44%), NO_x_ (13%–38%), and CO (11%–18%), with isoprene contributing 4%–14%. By contrast, in the pristine scenario represented by the Amazonas in Brazil [7], OH production is primarily governed by the O(^1^D) + H_2_O pathway, contributing up to 69%. Due to higher isoprene levels under pristine biogenic conditions, OH loss is dominated by reactions with isoprene, comprising 56% of total OH sinks in Amazon rainforest. These results indicate a clear transition in OH budget dynamics: in the present-day atmosphere, radical recycling via HO_2_ + NO serves as the principal production mechanism, while reactions with anthropogenically influenced species, i.e., VOCs, NO_x_ and CO, drive OH removal. In contrast, under pristine condition, primary photolytic sources and biogenic sinks, particularly isoprene, controlled OH production and loss.

### Turning points of OH concentrations across three regions

The dependence of OH concentrations on NO_2_ reveals a nonlinear response characterized by a distinct turning point near 3 ppb (Fig. 4). When NO_2_ concentrations are below this threshold, i.e., representing the NO_x_-limited regime, OH concentrations increase with rising NO_2_. Conversely, when NO_x_ exceeds ∼3 ppb, corresponding to the NO_x_-saturated regime, OH concentration decreases with further NO_2_ enhancement. From preindustrial period to 1950 or 1960, NO_2_ concentration remained largely within 0.001–3 ppb range, placing OH production on the ascending limb of the response curve. During this phase, increasing anthropogenic NO_x_ emissions drove a rise in OH concentrations. However, over the period 1985–2023, as NO_2_ levels exceeded 3 ppb, OH concentrations entered the descending limb of the curve, wherein reduction in NO_x_ emissions is associated with increases in OH (Fig. 2a–c). This regime shift is consistent with previous regional analysis in Guangdong Province [10].

A regionally resolved analysis of OBM-derived OH as a function of NO_2_ confirms the presence of well-defined turning points in both the United States and Europe (Fig. 4). Between 1985 and 2023, approximately 10% of observational stations in these regions were located within the NO_x_-limited regime, while ∼85% of the stations were situated within the NO_x_-saturated regime, indicating that recent NO_x_ reductions have likely enhanced OH levels. In contrast, no distinct turning point was observed in China during 2015–2023 and all cities were located within the NO_x_-saturated regime; OH concentrations there contribute to an increase with decreasing NO_2_.

The temporal evolution of the OH turning point has received limited attention in previous analyses. With increasing anthropogenic emissions during the mid-20^th^ century, the United States and Europe likely reached the OH turning point, marking the transition between NO_x_-limited and NO_x_-saturated regimes, as early as the 1950s to 1960s, subsequently entering the NO_x_-saturated regime (Fig. S6b). In China, the turning point likely occurred in the 1980s, with recent evidence suggesting a gradual shift back toward but not yet reaching NO_x_-limited regime. Over the past three decades, declining anthropogenic NO_x_ emissions have reversed this trend in all three regions, with OH concentrations beginning to respond accordingly. In the United States and Europe, approximately 10% of monitoring sites have already transitioned back into the NO_x_-limited regime. In contrast, China remains within the NO_x_-saturated regime and is projected to require an additional several decades to return fully to the NO_x_-limited regime. Similar trajectories are anticipated for regions with higher current pollution burdens, such as India and Southeast Asia, where the transition to NO_x_-limited regimes may require up to five decades under sustained emission reductions. From a hemispheric mid-latitude perspective, atmospheric surface OH concentrations have remained near a peak of plateaued close to the turning point, within the range of (5–9) × 10^6^ cm^−3^ over the past 50–60 years. Projections suggest that a more definitive shift back to the NO_x_-limited regime may not occur for another several decades.

### Uncertainty analysis

This study derives surface atmospheric OH concentrations based on the OBM method, which inevitably has certain limitations and uncertainties. First, uncertainties primarily arise from the simplified treatment of OH and NO_2_ source and sink terms in the continuity equation. The formation, consumption, and cycling processes of OH in the atmosphere are extremely complex. However, the OBM method does not fully account for the comprehensive effects of various substances, such as VOCs, O_3_, and HONO, that may regulate OH levels. This constitutes one of the significant sources of uncertainty in the estimation of OH. Additionally, we assume that the reactions of OH with NO_2_/NO_x_ are the primary loss pathways for NO_2_/NO_x_, and these are incorporated as the sole sink terms into Eq. 7. Other processes contributing to NO_2_/NO_x_ loss, such as photolysis of NO_2_/NO_x_, reactions with O_3_, RO_2_, and NO_3_, as well as aerosol uptake, precipitation, and dry deposition, are not further considered. These NO_2_ sinks would introduce significant uncertainty into our resultant OH values if the sinks are comparable to the oxidation of NO_2_ by OH. However, in our previous studies, we analyzed most of the sinks of NO_2_ mentioned above using both box models and a three-dimensional model [10,37]. Furthermore, heterogeneous reactions of NO_x_ are not accounted for, and the OH overestimation between 8:00 and 10:00 am is likely attributable to the influence of heterogeneous reactions.

The second significant uncertainty stems from the selection criterion of Lagrangian air mass. The selection criterion, defined by 80% of 1 standard deviation (1.0 ± 0.8σ) from the mean CO, NO_2_ distribution works well in filtering out those data deviating significantly from the Lagrangian condition. However, it also filters out approximately 65%–72% of the observational data, thereby limiting the representativeness of the OBM results to some extent. To assess this impact, we further applied selection criteria of 1.0 ± 0.5σ and 1.0 ± 1.0σ, which exclude about 80%, 56% of the data, respectively. The results under different selection criteria did not exhibit significant differences, indicating that the OBM analysis are reasonably representative for most samples. Additionally, applying the same dilution factor to both NO_2_ and CO may introduce further uncertainty.

Emission rate estimates also contribute to uncertainty. In this study, emission rates were calculated by grouping data into five-year intervals, under the assumption that hourly emission rates remained constant both within each five-year period and across daily hours of each season. We also assumed constant emission rates in the daytime, negligible dilution (Dil∼0) after the turning point (15:00 or 16:00 LT), and negligible OH concentrations (OH∼0) near sunset (17:00–18:00 LT in spring, 18:00–19:00 in summer, and 16:00–17:00 in autumn and winter). However, in urban cities, CO and NO_x_ emissions typically increase significantly during morning and evening traffic rush hours. Furthermore, pollutant emissions have decreased due to emission‑reduction policies. Therefore, directly attributing the observed changes in CO and NO_2_ concentrations before sunset to emission rates, while neglecting substantial diurnal and annual variations in emissions, as well as the effects of dilution and OH, likely leads to overestimated emissions rates. This overestimation could, in turn, result in overestimated hourly OH concentrations and obscure its true long‑term trend.

### Implications

Overall, mid-latitude OH levels in the atmosphere surface layer have hovered around the peak or turning point over the past 50–60 years, and it may take another several decades to definitively return to the NO_x_-limited regime. Variability in OH trends have direct implications for air quality control, of which secondary pollutants (e.g., O_3_, PM_2.5_) is predominantly produced via reactions of OH with primary pollutants (e.g., SO_2_, NO_x_, VOCs, etc.). Atmospheric oxidation capacity is intrinsically linked to secondary pollution formation. Mechanistically, the ozone production rate (P(O_3_)) can be parameterized as the product of the primary hydroxyl radical production rate (P(RO_x_)) and the OH chain length (ChL) [38]. During the COVID-19 lockdown, reductions in NO_x_ and other pollutant emissions in eastern China led to increases in both ozone production and atmospheric oxidizing capacity, thereby enhancing the formation of secondary fine particulate matter [39]. However, when extending the analysis to the entire Northern Hemisphere, decreases in NO_x_ emissions during the lockdown led to concurrent reductions in OH and O_3_ production [40]. Research shows that OH concentration and P(O_3_) respond nonlinearly to varying NO levels in both forested and urban areas [41]. Meanwhile, positive feedback exists between O_3_ and OH: the oxidation of VOCs drives O_3_ production, and O_3_ in turn photolyzes to generate OH, thereby accelerating further oxidation. Long-term observations in urban Beijing reveal a coherent increasing interannual trend in surface OH and ozone concentrations [18]. During 2006–2020, ozone production and total OH turnover rate were predominantly governed by a VOC-limited regime, with a discernible shift toward a transitional regime (between VOC-limited and NO_x_-limited regimes). Another study also shows that the dependence of P(O_3_) on NO_x_ is similar to that of OH on NO_x_ [42]. At high VOC reactivity and low NO_x_, both P(O_3_) and OH increase with rising NO_x_, characterizing a NO_x_-limited regime. Conversely, in NO_x_-saturated environments, P(O_3_) and OH show an inverse relationship with NO_x_. The interannual relationship between OH and NO_x_ can serve as a clear indicator for monitoring shifts in chemical regimes and provides important guidance for the development of emission control strategies aimed at reducing ozone pollution. At the regional scale, a study inferred tropospheric OH based on satellite CO observations and found that, OH in the Northern Hemisphere between 30°N and 60°N decreased by about 0.5% in 2020 relative to 2018–2019, whereas OH declined by 3%–8% in the region spanning 30°N to 60°S [40]. This suggests that atmospheric OH in the 30°N–60°N region is likely near a turning point, while OH in the equatorial and Southern Hemisphere regions may already be in the NO_x_-limited regime. This is broadly consistent with our conclusion that OH in mid-latitude region is reaching a turning point.

Our results suggest that, under continued anthropogenic NO_x_ mitigation, OH concentrations in the United States and Europe may be approaching a turning point in the steady-state concentration of OH, moving from NO_x_-saturated conditions to NO_x_-limiting conditions, while OH concentrations in China are still in NO_x_ saturated conditions. Recent studies have further indicated that different NO_x_ reduction strategies among countries have directly led to pronounced spatial heterogeneity in the OH trends [41]. Similar to the trend in OH concentration, O_3_ concentrations in Europe and the United States have begun to decline since 1980, while in China they continue to rise [43,44]. This reflects a different turning point in the O_3_ trend between China and Western countries. The OH trend has implications for ozone control strategies, as it suggests that if current control strategies continue, ozone production in the United States and Europe will soon transition into NO_x_-limited conditions while China may not reach this transition for several more decades, likely requiring additional VOC controls during this period. These results suggest the necessity of incorporating dynamic changes in atmospheric oxidation capacity into regional air quality improvement strategies targeting anthropogenic emissions.

## METHODS

### OH calculation

Previous studies have demonstrated that the ratio of ethylbenzene and m, p-xylene, emitting from common sources but exhibiting differing reactivity with OH, can serve as a proxy for photochemical oxidation driven by OH [23]. If we follow a Lagrangian trajectory and consider the influence of transport, then:


(1) \begin{eqnarray*}
E( t) = E( 0)\exp \left( { - {k}_e\int_{0}^{t}{{\left[ {\rm OH} \right]}}\ dt} \right) \times {F}_1\left( {\textit{Transport}} \right),
\end{eqnarray*}

(2) \begin{eqnarray*}
X( t) = X( 0)\exp \left( { - {k}_x\int_{0}^{t}{{\left[ {\rm OH} \right]}}\ dt} \right) \times {F}_2\left( {\textit{Transport}} \right),
\end{eqnarray*}

where *E*(*t*) and *X*(*t*) represent concentrations of ethylbenzene and m, p-xylene at time *t*, respectively. *E*(0) and *X*(0) are their corresponding initial concentrations, *k_x_* and *k_e_* are their reaction rate constants with OH, and *k_x_* and *k_e_* equal to 2.17 × 10^–11^ and 7.0 × 10^–12^ cm^3^ s^–1^, respectively. Because *E* and *X* have same sources and sinks, they should be affected by the same transport process, i.e., *F_1_ = F_2_*. Thus:


(3) \begin{eqnarray*}
E( t)/X( t) &=& E( 0)/X( 0)\\
&&\times \exp \left( { - \int_{0}^{t}{{\left( {{k}_e - {k}_x} \right)\left[ {\rm OH} \right]}}\ dt} \right).\\
\end{eqnarray*}

The OH concentrations are evaluated using CO and NO_2_ instead of ethylbenzene and m, p-xylene, primarily due to the lack of measurements of VOCs [10,37]. Derivation of OH concentrations based on CO and NO_2_ observed along a Lagrangian trajectory is accomplished as follows:


(4) \begin{eqnarray*}
\left[ {{\rm NO}_2} \right]{\mathrm{ = }}\,{\left[ {{\rm NO}_2} \right]}_0\exp \left( { - {k}_4\int\nolimits_{0}^{t}{{\left[ {\rm OH} \right]}}dt} \right),
\end{eqnarray*}

where *k*_4_ is the reaction rate constant of NO_2_ with OH. The value of *k*_4_ is 1.04 × 10^−11^ cm^3^ s^–1^ at 25°C and 1 atm pressure [45].

In this study, an improved OBM method for calculating OH is based on the continuity equation of CO and NO_2_. Taking Guangzhou as an example, the hourly CO concentrations in each season exhibit an inflection point in the afternoon (Fig. S1a). Therefore, the continuity equation for CO can be expressed as:


(5) \begin{eqnarray*}
\frac{{dN}}{{dt}} = P - L - \Delta \textit{Flux},
\end{eqnarray*}

where *N* represents the concentration of CO at a given time *t. P* represents the emission rate (ppb/h) of CO (*Em*_co_), while *L* represents the chemical loss of CO, which is negligible. Δ*Flux* signifies the dilution of CO (*Dil*_co_). Consequently, the equation can be simplified to:


(6) \begin{eqnarray*}
\frac{{d}{\rm CO}}{{dt}} = E{m}_{\rm CO} - Di{l}_{\rm CO}.
\end{eqnarray*}

Similarly, for NO_2_, as depicted in Fig. S1b, we observe the following equation:


(7) \begin{eqnarray*}
\frac{{d}{\rm NO}_2}{{dt}} = E{m}_{{\rm NO}_2} - {k}_4 \times {\rm OH} \times {\rm NO}_2 \times t - Di{l}_{{\rm NO}_2}.
\end{eqnarray*}

The dilution of CO and NO_2_ is calculated as follows:


(8) \begin{eqnarray*}
Di{l}_{\rm CO} = E{m}_{\rm CO} - \frac{{d{\rm CO}}}{{dt}},
\end{eqnarray*}

(9) \begin{eqnarray*}
Di{l}_{{\rm NO}_2} = Di{l}_{\rm CO} \times \frac{{\left[ {{\rm NO}_2} \right]}}{{\left[ {\rm CO} \right]}}.
\end{eqnarray*}

The seasonal emission rates of CO and NO_2_ at each city in China are calculated individually using station data from 2015 to 2023, divided into five-year intervals (2015–2019 and 2020–2023) at the city level, each further divided by season. As an example, Fig. S1 shows the diurnal profiles of CO and NO_2_ in Guangzhou across four seasons during 2015–2019. In these profiles, the transitions near the turning points between 10:00 and 19:00 LT are not smooth, introducing uncertainty into emission estimates. To address this, we performed additional fitting around these turning points. We assumed constant emission rates in the daytime, negligible dilution (Dil∼0) after the turning point, and low OH levels (OH∼0) before sunset (spring: 17:00–18:00 LT; summer: 18:00–19:00 LT; autumn/winter: 16:00–17:00 LT). Under these assumptions, the change in the fitting curves during this period was taken as the emission rate of CO and NO_2_. For the United States and Europe, CO and NO_2_ observations at each station from 1985 to 2023 were grouped into available five‑year intervals (e.g., 1985–1989, 1990–1994, …, 2020–2023), with incomplete records grouped according to actual data coverage. Each interval was then subdivided by season. Seasonal emission rates were estimated using the same method applied in China, based on seasonal diurnal variations.

Following the estimation of OH concentrations from hourly CO and NO_2_ observations, a data filtering procedure was applied to ensure consistency with Lagrangian transport assumptions (see Text S2).

### Chemical box model

A zero-dimensional chemical box model based on the RACM2–LIM1 mechanism was used to simulate OH, HO_2_, and RO_2_ concentrations under observational constraints, following protocols established in prior field campaign [24,46,47]. The RACM was modified by replacing its isoprene mechanism with the more detailed mechanism presented in Table S2 and previous study [24]. The impact of the isoprene oxidation mechanism, which replaces the original isoprene chemistry in RACM2, on OH simulations is also presented in Table S2. In this study, the model was applied to simulate OH budgets under present-day scenario at three representative stations, i.e., Shanghai (China), New York (the United States), and Jülich (Germany). Model inputs included observational data for O_3_, CO, NO_2_, NO, SO_2_, VOCs, HONO, photolysis frequencies, relative humidity, solar zenith angle, ambient temperature, and pressure. Due to the absence of HONO measurements during the Shanghai campaign, HONO concentrations were parameterized as 2% of the observed NO_2_ concentration, consistent with constant ratios reported in previous studies [24,48,49]. The associated uncertainties introduced by this parameterization are discussed in the cited literature. A first-order loss term equivalent to a 6-hour lifetime was applied uniformly to all species to represent physical removal processes including deposition, convection, and advection. A spin-up period of three days was used to ensure steady-state conditions for unconstrained species. Details descriptions of the measurement sites and parameterized inputs are provided in Tables S1 and S3. For the preindustrial scenario with pristine atmosphere, due to the uncertainty associated with estimating both VOC and NO_x_ concentrations, its OH budget is adopted by the simulation of Amazonas measurement in Brazil, a typical pristine environment with minimal anthropogenic emissions (OH budget from model run embedded with RACM2 mechanism in Fig. 8d in [7]).

The OH radical budget is governed by a nonlinear interaction among initiation processes (e.g., photolysis of closed-shell precursors), chain propagation steps (e.g., interconversion between RO_2_, HO_2_, and OH), and termination reactions leading to stable end products. Among these, OH production via reactions of RO_2_ and HO_2_ with NO_x_ plays a dominant role across a wide range of NO_x_ concentrations.

To examine the response of OH to varying NO_2_ levels, simulations were conducted using NO_2_ as a variable input from 0.2 and 100 ppb, while other inputs, i.e., O_3_, CO, SO_2_, CH_4_, VOCs, HONO, photolysis frequency, relative humidity, solar zenith angle, ambient temperature, and pressure, were constrained to their noontime (11:00–13:00 LT) averages. For the low-NO_2_ regime (0.001–0.2 ppb), all variables except O_3_, CO, CH_4_ and isoprene were fixed at their noontime averages. O_3_, CO, and isoprene were derived from field observations in the Amazon rainforest, served as pristine atmosphere due to minimal anthropogenic emissions [7]. CH_4_ concentrations were based on the global atmospheric mean mixing ratio from the recommended CMIP5 dataset, which incorporates Law Dome ice core measurements compiled by Etheridge *et al.* (1998) [50]. According to previously published parameterizations, we further assumed a linear relationship between CH_4_ and NO_2_, and therefore derived CH_4_ concentration ranges via linear interpolation based on observed NO_2_ variations. The corresponding values are provided in Table S4. A 10-hour spin-up time was used in this regime to ensure that unconstrained species reached a steady state.

## Supplementary Material

nwag178_Supplemental_File

## Data Availability

Hourly ground-based observations of O_3_, NO_2_ and CO were obtained from the China National Environmental Monitoring Centre (http://www.cnemc.cn/en/), the United States Environmental Protection Agency (https://aqs.epa.gov/aqsweb/airdata/download_files.html#Raw), and the European Environment Agency (https://eeadmz1-downloads-webapp.azurewebsites.net/), respectively. Annual anthropogenic nitrogen dioxide emissions were obtained from the Community Emissions Data System (CEDS) (https://zenodo.org/records/12803197, https://visualizingenergy.org/global-anthropogenic-nitrogen-dioxide-emissions-1750-2022/). This data set uses existing emission inventories, emission factors, and activity/driver data to estimate annual country-, sector-, and fuel-specific emissions over time [51].
