# Supplementary material for: Atmospheric oxidation capacity at northern mid-latitude regions is reaching a turning point
Source: Natl Sci Rev. 2026 Mar 24;13(8):nwag178. doi: 10.1093/nsr/nwag178 (PMC13156942; doi:10.1093/nsr/nwag178)
Supplement: nwag178_Supplemental_File [file nwag178_supplemental_file.pdf]

Supplementary Data for

Atmospheric oxidation capacity at northern midlatitude regions is reaching a turning point

Liyan Wei[1], Shaw Chen Liu[2], Zhaofeng Tan[1], Xuefei Ma[1], Ming Zhou[1], Wenyu Wei[1], Jingwen Zhang[2], Yuanhang Zhang[1], Run Liu[2,*] and Keding Lu[1,*]

[1] State Key Laboratory of Regional Environment and Sustainability, College of Environmental Sciences and Engineering, Peking University, Beijing 100871, China

[2] Institute for Environmental and Climate Research, College of Environmental and Climate, Jinan University, Guangzhou 511443, China

* Corresponding authors: Run Liu (liurun@jnu.edu.cn) and Keding Lu (k.lu@pku.edu.cn)

This PDF file includes:

Glossary

Supplementary Texts 1 to 2

Supplementary Tables 1 to 5

Supplementary Figures 1 to 7

References

Glossary

OH	hydroxyl radical
ISO	Isoprene
HCHO	Formaldehyde
ACE	Acetylene
ETE	Ethene
ETH	Ethane
HC3	Alkanes, esters and alkynes with HO rate constant (298 K, 1 atm) less than $3.4x10^{-12}$ cm^3 s^{-1}
HC5	Alkanes, esters and alkynes with HO rate constant (298 K, 1 atm) between $3.4x10^{-12}$ and $6.8x10^{-12}$ cm^3 s^{-1}
HC8	Alkanes, esters and alkynes with HO rate constant (298 K, 1 atm) greater than $6.8x10^{-12}$ cm^3 s^{-1}
OLI	Internal alkenes
OLT	Terminal alkenes
BEN	Benzene
TOL	Toluene and less reactive aromatics
XYO	o-xylene
XYP	P-xylene
XYM	M-xylene
MOH	Methanol
ACT	Acetone
MEK	Methyl ethyl ketone
HKET	Hydroxy ketone
UALD	Unsaturated aldehydes
LIM	d-limonene and other cyclic diene-terpenes
BALD	Benzaldehyde and other aromatic aldehydes
KET	Ketones
MGLY	Methylglyoxal and other alpha-carbonyl aldehydes
MVK	Methyl vinyl ketone
MACR	Methacrolein
CSL	Cresol and other hydroxy substituted aromatics
API	Alpha-pinenes and other cyclic terpenes with one double bond
ISOP	Isoprene peroxy radical
ppb	Parts per billion in a volume fraction
ppt	Parts per trillion in a volume fraction
OH + ISO	the oxidation rate by reaction of OH with isoprene

Supplementary Text

1. Data quality control for the hourly raw data

To ensure data completeness and reduce the influence of missing observations, strict quality control procedures were applied to the measurement datasets of China, the United States and Europe. For each station, daily NO_2 data were retained only when at least 20 hourly measurements were available. A given month was included in the analysis only if it contained data from more than 15 such days, and a year was retained only if it included a minimum of eight valid months. To ensure consistency, CO data were considered valid for a given year only if they covered at least 80% of the valid NO_2 observations. Stations that failed to meet these criteria were excluded from the analysis. Annual average concentrations were computed based on the filtered hourly datasets.

The numbers of NO_2 hourly data at each day (ND) at a specific monitoring site are marked as valid and retained if they meet all of the following criteria:

(1) ND is at least 20, i.e. ND $\geq$ 20;

(2) The numbers of ND $\geq$ 20 at each month (NM) are at least 15, i.e. NM $\geq$ 15;

(3) The numbers of NM $\geq$ 15 at each year (NY) are at least 8, i.e. NY $\geq$ 8;

(4) The numbers of CO hourly data (CH) are then accounted for at least 80% of the NO_2 data (NH) at each year, i.e. CH $\geq$ 80% NH.

2. The Lagrangian condition

By combining the aforementioned equations, hourly OH concentrations can be estimated using hourly observations of CO and NO_2. The Lagrangian condition applied here refers to a steady air mass, that is, the air mass at time t and the air mass at time ($t+1$) are from the same air mass. Since the Lagrangian conditions are not always observed, it is necessary to make a selection criterion to filter out data that deviate significantly from the quasi-Lagrangian condition as shown in Fig. S1. The selection criterion is that the CO and NO_2 concentrations at time t lie within 80% of 1 standard deviation (vertical bars on Fig. S1) of the CO and NO_2 average values (black spot) at time t, respectively, which is assumed to be in the Lagrangian condition. As illustrated in Fig. S1, CO and NO_2 concentrations exhibit highly similar diurnal variations patterns across different seasons, indicating that the air masses corresponding to the hourly concentrations of CO and NO_2 along the curve are likely of common sources. This consistency can be considered to satisfy the Lagrangian conditions. Consequently, applying the aforementioned criteria for data selection allows for the retention of enough number of data points along the curve and in its vicinity, while effectively

excluding interferences from transport, heterogeneous air mass sources and conditions such as precipitation, cloudy weather, and fog. Moreover, OH outliers below 0 or excessively large (calculated OH more than 10-fold higher than the average OH concentration at each city or station) will be excluded. These criterions filter out (65–72)% of data; i.e., approximately 32% of the days satisfy approximately the Lagrangian condition. We have tested this selection criterion by parameterizing it between 50% and 100% of 1 standard deviation and found our major results are robust within this range[1].

Table S1. Location information of measurement sites.

Site	Location	Observation period	References
Shanghai	121.54° E, 31.12° N	28 Aug–10 Sep 2016	This study
New York	73.8° W, 40.7° N	30 Jun–03 Aug 2001	ref[2]
Jülich	6.4° E, 50.9° N	24 Aug–01 Sep 2019	ref[3]

Table S2. The impacts of isoprene chemistry used in RACM2 and RACM2–LIM1 for OH simulation in present periods.

Site	Differences (RACM2–LIM1 vs RACM2)	
Shanghai	OH	1%
	OH budget	1%
New York	OH	0%
	OH budget	0%
Jülich	OH	6%
	OH budget	7%
Isoprene oxidation scheme	**RACM2**: (1) ISO + OH → ISOP<br>**RACM2–LIM1**:<br>(1) ISO + OH → ISOP<br>(2) ISOP = MACR + HCHO + OH<br>(3) ISOP = MVK + HCHO + OH<br>(4) ISOP = HPALD1 + HO2 + HPCARPO2<br>(5) ISOP + NO = HPALD1 + HO2 + HPCARPO2 + NO<br>(6) ISOP + HO2 = HPALD1 + HO2 + HPCARPO2 + HO2<br>(7) ISOP + ACO3 = HPALD1 + HO2 + HPCARPO2 + ACO3<br>(8) ISOP + MO2 = HPALD1 + HO2 + HPCARPO2 + MO2<br>(9) ISOP = HPALD2 + HO2 + HPCARPO2<br>(10) ISOP + NO = HPALD2 + HO2 + HPCARPO2 +NO<br>(11) ISOP + HO2 = HPALD2 + HO2 + HPCARPO2 +HO2<br>(12) ISOP + ACO3 = HPALD2 + HO2 + HPCARPO2 +ACO3<br>(13) ISOP + MO2 = HPALD2 + HO2 + HPCARPO2 +MO2<br>(14) HPALD1 = OH + HO2 +0.5HKET + 0.5MGLY + 0.5ALD + HCHO<br>(15) HPALD2 = OH + HO2 + 0.5HKET + 0.5GLY + 0.5ALD + HCHO<br>(16) HPALD1 + OH = OH<br>(17) HPALD2 + OH = OH<br>(18) HPCARPO2 = CO + OH + OP2<br>(19) HPCARPO2 + NO = NO2 + MGLY + OH + OP2 + PO3<br>(20) HPCARPO2 + HO2 = OP2<br>(21) ISHP + OH = IEPOX + OH<br>(22) ISHP + OH = 0.7ISOP +0.3MACR + 0.3OH<br>(23) IEPOX + OH = IEPOXO2<br>(24) IEPOXO2 + NO = IEPOXO + NO2 + PO3<br>(25) IEPOXO2 + HO2 = IEPOXO + OH + O2<br>(26) IEPOXO = 0.125OH + 0.825HO2 + 0.251CO + 0.725HKET +	

0.275GLY + 0.275ALD + 0.074ORA1 + 0.275MGLY + 0.375HCHO

(27) MCP = HKET + OH + CO

(28) MACP + NO = 0.65MO2 + 0.65CO + 0.35ACO3 + NO2 + HCHO + PO3

(29) MCP + NO = N2 + HO2 + HKET + CO

(30) MVKP + HO2 = OP2

(31) MVKP + HO2 = ACO3 + OH + ALD

(32) MVKP + HO2 = HO2 + OH + ORA2

Table S3. Noontime (11:00–13:00 LT) average values of input parameters used in the box model at various measurement sites for NO_2 concentrations in the range of 0.2–100 ppb.

Parameter	Shanghai	New York	Jülich
O_3 (ppb)	89.4	41.6	56.6
CO (ppb)	593	314	139
SO_2 (ppb)	4.6	——	——
NO (ppb)	1.3	7.3	0.2
NO_2 (ppb)	10.1	17.8	2.3
CH_4 (ppb)	——	1925	1956
HONO (ppb)	——	0.377	0.229
HCHO (ppb)	——	5.900	3.100
ISO (ppb)	0.558	0.226	0.275
ACE (ppb)	0.255	——	——
ETE (ppb)	0.256	——	——
ETH (ppb)	1.054	——	——
HC3 (ppb)	4.600	3.355	——
HC5 (ppb)	1.584	1.677	——
HC8 (ppb)	1.143	0.659	——
OLI (ppb)	0.179	0.354	0.005
OLT (ppb)	0.710	0.821	0.023
BEN (ppb)	0.409	——	0.036
TOL (ppb)	1.987	1.884	0.072
XYO (ppb)	0.177	0.209	0.007
XYP (ppb)	0.066	0.193	0.006
XYM (ppb)	0.673	0.123	0.004
MOH (ppb)	——	——	3.635
ACT (ppb)	——	——	2.198
MEK (ppb)	——	——	0.178
HKET (ppb)	——	——	0.213

UALD (ppb)	——	——	0.055
LIM (ppb)	——	——	0.013
BALD (ppb)	——	——	0.008
KET (ppb)	——	——	0.073
MGLY (ppb)	——	——	1.286
MVK (ppb)	——	——	0.178
MACR (ppb)	——	——	0.089
CSL (ppb)	——	——	0.004
Photolysis frequencies (s^{-1})			
$O_3 \rightarrow O^1D$	1.5×10^{-5}	2.3×10^{-5}	0.8×10^{-5}
$NO_2 \rightarrow NO + O$	4.3×10^{-3}	7.0×10^{-3}	3.2×10^{-3}
$HONO \rightarrow NO + OH$	——	1.1×10^{-3}	0.5×10^{-3}
$H_2O_2 \rightarrow 2OH$	7.0×10^{-6}	1.2×10^{-6}	——
$HCHO \rightarrow H_2 + CO$	4.3×10^{-5}	3.5×10^{-5}	1.7×10^{-5}
$HCHO \rightarrow 2HO_2 + CO$	2.9×10^{-5}	2.3×10^{-5}	1.0×10^{-5}
$NO_3 \rightarrow O_2 + NO$	——	——	0.090
$NO_3 \rightarrow O\left(^3P\right) + NO_2$	——	——	0.012
Solar zenith angle (°)	27.7	25.2	43.9
Relative humidity (%)	65.3	28.5	34.8
Temperature (°C)	26.2	28.6	30.4
Pressure (hPa)	1007.4	1013.0	1004.7

Table S4. Noontime (11:00-13:00 LT) average values of input parameters used in the box model at various measurement sites for NO_2 concentrations in the range of 0.001–0.2 ppb.

Parameter	Shanghai	New York	Jülich
NO_2 (ppb)	0.001–0.2	0.001–0.2	0.001–0.2
O_3 (ppb)	17	17	17
CO (ppb)	130	130	130
CH_4 (ppb)	500–1500	500–1500	500–1500
ISO (ppb)	2	2	2

Table S5. General information of the OH concentration from various field campaigns under low NO_2 and high NO_2 environments.

Classification	Campaigns	Local time	NO_2(ppt)	OH ($\times10^6$ cm^{-3})	Location	References
Forest (B)	GoAmazon (Feb–Mar 2014)	10:00–15:00	85	1.0±0.6	Amazonas, Brazil (3.1° S, 60.6° W)	ref[4]
Forest (B)	OP3 (Apr–May 2008)	10:00–12:00	130	2.2	Malaysia (5.0° N, 117.8° E)	ref[5]
Forest (B)	BEARPEX09 campaign (Jun–Jul 2009)	9:40–17:10	180	1.5±0.3	Ponderosa pine plantation, in the California Sierra Nevada Mountains (38.9° N, 120.6° W)	ref[6]
Forest (B)	BEARPEX09 (Jun–Jul 2009)	09:00-15:00	200	1.3	California Sierra Nevada Mountains (38.9° N, 120.6° W)	ref[6]
Forest (B)	CABINEX (Jul–Aug 2009)	11:00–14:00	220	1.3	Deciduous forest in northern Michigan (44.8° N, 84.8° W)	ref[7]

Forest (B)	PROPHET–AMOS (Summer 2016)	11:45–16:15	250	1.2±0.2	Midlatitude deciduous broadleaf forest, in northern Michigan (45.56° N, 84.71° W)	ref[8]
Forest (B)	SOAS (Jun–Jul 2013)	10:00–15:00	293	1.2	Talladega National Forest in Brent, Alabama (32.9° N, 87.2° W)	ref[9]
Forest (B)	PROPHET (August 1998)	10:00–11:00	456	3.6	Deciduous forest in northern Michigan (45.6° N, 84.7° W)	ref[10]
Ocean (A)	Coastal Antarctica (summer 2005)	06:00–18:00	5	0.4±0.2	British Antarctic Survey's Halley Research Station (75.58° S, 26.32° W)	ref[11]
Ocean (A)	OOMPH project (March 2007)	11:00–13:00	14	3.1±2.2	Southern Atlantic Ocean, at the French research vessel Marion-Dufresne (28° S–57° S, 46° W–34° E)	ref[12]
Ocean (A)	RHaMBLe project (May–Jun 2007)	11:00–13:00	16	5.0±2.9	Cape Verde Atmospheric Observatory (16.85° N, 24.87° W), located in the tropical Atlantic	ref[13]

					marine boundary layer	
Ocean (A)	Seasonal Oxidant Study for SOS1 (Feb–Mar 2009)	11:00–13:00	55	2.1±1.3	Cape Verde Atmospheric Observatory (16.85° N, 24.87° W), located in the tropical Atlantic marine boundary layer	ref[14]
Ocean (A)	EASE97 (Apr–May 1997)	11:00–15:00	91	1.4	Mace Head, Ireland (53.3° N, 9.9° W)	ref[15]
Ocean (A)	INTEX-A (summer 2004, altitude 0 km)	—	98	4.2	Western Atlantic Ocean	ref[16]
Ocean (A)	EASE96 (Jul–Aug 1996)	Daily average	149	2.3	Mace Head, Ireland (53.3° N, 9.9° W)	ref[17]
Ocean (A)	RISFEX (September 2003)	09:00–15:00	208	2.0±0.5	Rishiri Island (45.1° N, 141.1° E), Japan	ref[18]
Ocean (A)	ORION99 Campaign (August 1999)	11:00–13:00	410	1.8±1.2	Remote marine site of Okinawa (26.9° N, 128.3° E)	ref[19]
Ocean (A)	Japan (summers of 1998–2000)	11:00–13:00	600	4.1±1.6	Oki Dogo Island (36.3° N, 133.2° E), Okinawa Island (26.9° N,	ref[20]

					128.3° E), and Rishiri Island (45.1° N, 141.1° E), Japan	
Rural areas (C)	Idaho Hill experiment (Aug–Sep 1993)	12:00–13:00	185	3.0±1.1	Idaho Hill, Colorado (39.9° N, 105.6° W)	ref[21]
Rural areas (C)	CYPHEX campaign (summer 2014)	13:00–16:00	293	4.3±2.6	Northwest coast of Cyprus (34.95° N, 32.38° E)	ref[22]
Rural areas (C)	Idaho Hill experiment (Aug–Sep 1993)	11:00–13:00	425	2.2±0.9	Idaho Hill, Colorado (39.9° N, 105.6° W)	ref[23]
Rural areas (C)	Fritz Peak Observatory (Jul–Aug 1991)	11:00–13:00	450	1.6±0.2	Fritz Peak Observatory, Colorado (39.9° N, 105.5° W)	ref[24]
Rural areas (C)	MINOS campaign (August 2001)	05:50–18:50	510	4.5±1.1	Finokalia Station of Crete, Greece (35.3° N, 25.7° E)	ref[25]
Rural areas (C)	FluCS 2021 (Summer 2021)	11:45–16:15	780	5.8±1.2	Woodland Park, Colorado (39.0° N, 105.05° W)	ref[26]

Rural areas (C)	DOMINO (Nov–Dec 2008)	11:00–14:00	1000	2.7±1.0	El Arenosillo, southern Spain (37.1° N, 6.7° W)	ref[27]
Cities (D3)	ICOZA campaign (July 2015)	11:00–13:00	1200	4.3±3.1	Coastal site (53.0° N, 1.1° E) in north Norfolk, UK	ref[28]
Cities (D3)	BERLIOZ (July/August 1998)	11:00–13:00	1530	3.4±1.5	Rural site Pabstthum about 50 km NW of Berlin (52.85° N, 12.94° E)	ref[29]
Cities (D1)	PRIDE-PRD2006 (July 2006)	12:00–15:00	1700	12.9	Backgarden site (23.5° N, 113.03° E), a rural environment in the Pearl River Delta, China	ref[30, 31]
Cities (D2)	TRAMP2006 (Aug–Sep 2006)	11:00–13:00	2030	11.2	University of Houston campus (29.72° N, 95.34° W)	ref[32]
Cities (D1)	OH measurements (Oct–Nov 2020)	11:00–13:00	2400	4.8±2.5	Hok Tsui (HT) (22.21° N, 114.25° E), coastal site in Hong Kong	ref[33]
Cities (D3)	ICOZA (July 2015)	11:00–13:00	2500	8.0±3.6	Coastal site in north Norfolk, UK (52.95° N, 1.12° E)	ref[34]

Cities (D3)	WAOSE95 (June 1995)	12:00–13:00	2500	6.0±1.8	Weybourne, UK (53° N, 1° E)	ref[35]
Cities (D1)	CAREBEIJING2006 (Aug–Sep 2006)	12:00–15:00	3000	7.7	Yufa site (39.5° N, 116.3° E), a suburban environment in Beijing, China	ref[36]
Cities (D3)	MEGAPOLI summer field campaign (July 2009)	12:00–14:00	3000	4.4±1.3	SIRTA observatory near Paris, France (48.718° N, 2.207° E)	ref[37]
Cities (D1)	Wangdu (Jun–Jul 2014)	12:00–16:00	3300	6.9	Wangdu (38.7° N, 115.2° E), located in the North China Plain, China	ref[38]
Cities (D1)	EXPLORE-YRD campaign (May–Jun 2018)	11:00–13:00	4100	10.0±1.3	Suburban site (32.56° N, 119.99° E) in Yangtze River Delta, China	ref[39]
Cities (D2)	TEXAQS2000 (Aug–Sep 2000)	11:00–13:00	4250	17.1	La Porte, Texas, southeast of Houston (29.67° N, 95.02° W)	ref[32]
Cities (D3)	JULIAC campaign (Summer 2019)	10:00–12:00	4600	6.5±2.4	Forschungszentrum Jülich (FZJ, 50.9° N, 66.41° E), Germany	ref[3]
Cities (D2)	SHARP 2009 (Apr–Mar 2009)	10:45–14:15	5200	7.2±1.4	University of Houston (29.72° N, 95.34° W), in southeast Texas	ref[40]

Cities (D3)	HOxComp field campaign (July 2005)	11:00–13:00	5460	6.7±1.9	Forschungszentrum Jülich (FZJ, 50.9° N, 66.41° E), Germany	ref[41]
Cities (D1)	CHOOSE-2019 (Aug–Sep 2019)	11:00–13:00	6100	9.4±3.4	Suburban site (30.68° N, 103.85° E) in Chengdu, China	ref[42]
Cities (D1)	PRD (October 2019)	11:00–13:00	7000	7.5±2.2	Coastal continental site (22.55° N, 114.60° E) in the Pearl River Delta, China	ref[43]
Cities (D3)	ClearfLo campaign (Jul–Aug 2012)	11:00–13:00	7200	3.9±1.3	Sion Manning School in North Kensington (51.52° N, 0.21° W), in the central London	ref[44]
Cities (D1)	BEST-ONE campaign (Jan–Mar 2016)	11:00–13:00	7400	3.0±1.8	Suburban site Huairou near Beijing (40.41° N, 116.68° E)	ref[45]
Megacities (E)	IMPACT-L (Jul–Aug 2004)	11:00–13:00	9700	6.3	Komaba site (35.6 °N, 139.7 °E) on the University of Tokyo, Japan	ref[18]
Megacities (E)	STORM campaign (autumn of 2018)	11:00–13:00	10900	4.2±0.9	Shenzhen site (22.60° N, 113.97° E) in the Pearl River Delta	ref[46]
Megacities (E)	CalNex–LA campaign (May–Jun	11:00–13:00	11000	4.0±3.0	California Institute of Technology	ref[47]

	2010)				(34.13° N, 118.12° W) in Pasadena, Los Angeles	
Megacities (E)	Ozone episode in Beijing (Sep–Oct 2016)	11:00–13:00	12000	7.2±1.4	Peking University (39.99° N, 116.31° E) in Beijing, China	ref[48]
Megacities (E)	MCMA2003 (April 2003)	11:00–13:00	13000	8.9	Iztapalapa site (19.4° N, 99.2° W), in south-central Mexico City	ref[32]
Megacities (E)	PRD (autumn 2014)	10:00–14:00	14100	4.0±2.0	Heshan (22.7° N, 112.9° E), Guangdong	ref[49]
Megacities (E)	APHH campaign (Nov–Dec 2016)	11:00–13:00	15400	3.2	IAP site (39.97° N, 116.37° E) in Beijing, China	ref[50]
Megacities (E)	PMTACS-NY2001 (Jun–Aug 2001)	11:00–13:00	1580	7.7	US EPA host site (40.7° N, 73.8° W) on the campus of Queens College in the Borough of Queens, New York City	ref[32]
Megacities (E)	Beijing (AIRPRO) campaign (summer of 2017)	08:00–13:00	19200	6.8±1.7	IAP site (39.97° N, 116.37° E) in Beijing, China	ref[51]

Megacities (E)	SMART-2019 (Nov–Dec 2019)	11:00–13:00	19500	2.7±1.3	SAES site (31.17° N, 121.43° E) in downtown Shanghai	ref[52]
Megacities (E)	PKU campaign (November 2017 to January 2018)	11:00–13:00	21000	1.8±0.9	Peking University (39.99° N, 116.31° E) in Beijing, China	ref[53]
Megacities (E)	LAFRE (September 1993)	11:00–13:00	22500	4.7±0.4	Claremont (34.1° N, 117.7° W), downwind of Los Angeles, California	ref[54]
Megacities (E)	MCMA2006 (March 2006)	08:40–18:40	28400	3.2	Instituto Mexicano del Petroleo (19.5° N, 99.1° W), in the Mexico City Metropolitan Area	ref[55]

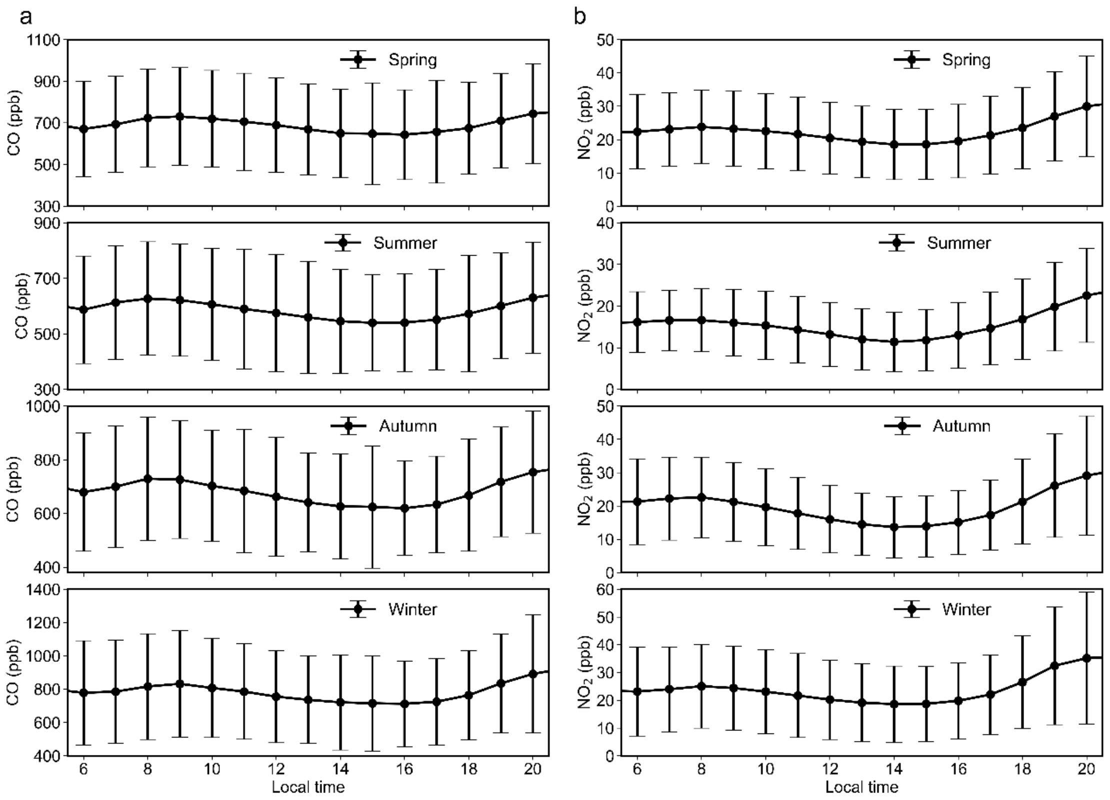

Figure S1. Diurnal variations (06:00–20:00 LT) over different seasons. CO (a) and NO_2 (b) concentrations in Guangzhou (2015–2019). Black spots with vertical bars represent the range of mean values ± the 1 standard deviation.

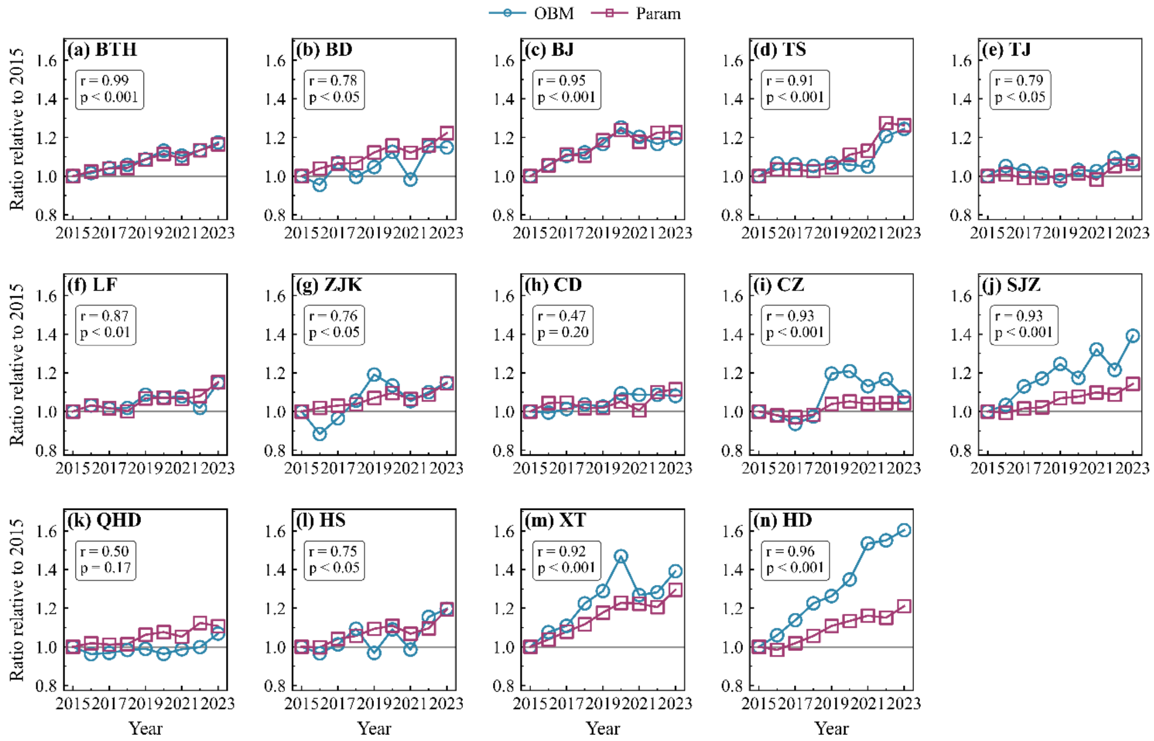

Figure S2. The ratio of OH concentrations in the Beijing–Tianjin–Hebei region (a, BTH) calculated based on OBM (blue circles) and empirical parametrization (purple boxes) during the 2015–2023 period compared to 2015. (b) Baoding (BD), (c) Beijing (BJ), (d) Tangshan (TS), (e) Tianjin (TJ), (f) Langfang (LF), (g) Zhangjiakou (ZJK), (h) Chengde (CD), (i) Cangzhou (CZ), (j) Shijiazhuang (SJZ), (k) Qinhuangdao (QHD), (l) Hengshui (HS), (m) Xingtai (XT), (n) Handan (HD).

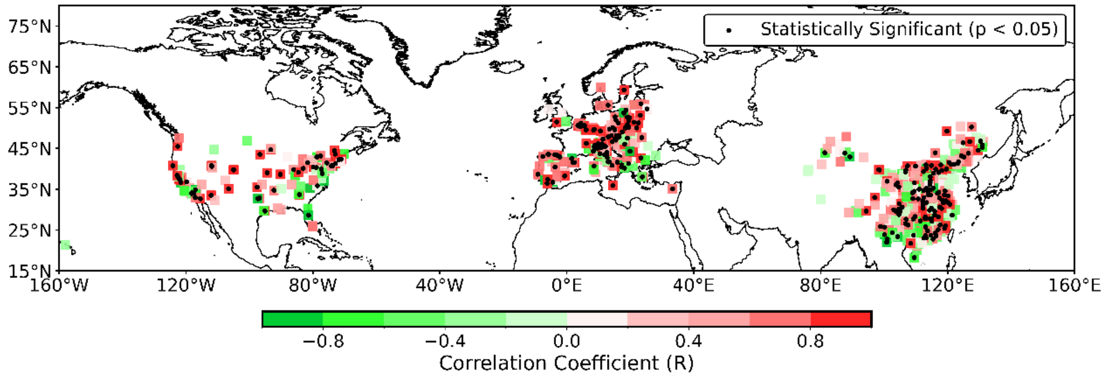

Figure S3. Spatial distributions of correlation coefficients. Trends of annual mean OH in China, the United States and Europe, respectively, for 2015–2023. Correlation coefficients (R) of the linear fits of the OH trends (red boxes with black dots represent significant positive correlations (*p<0.05*), and green boxes with black dots represent significant negative correlations (*p<0.05*)).

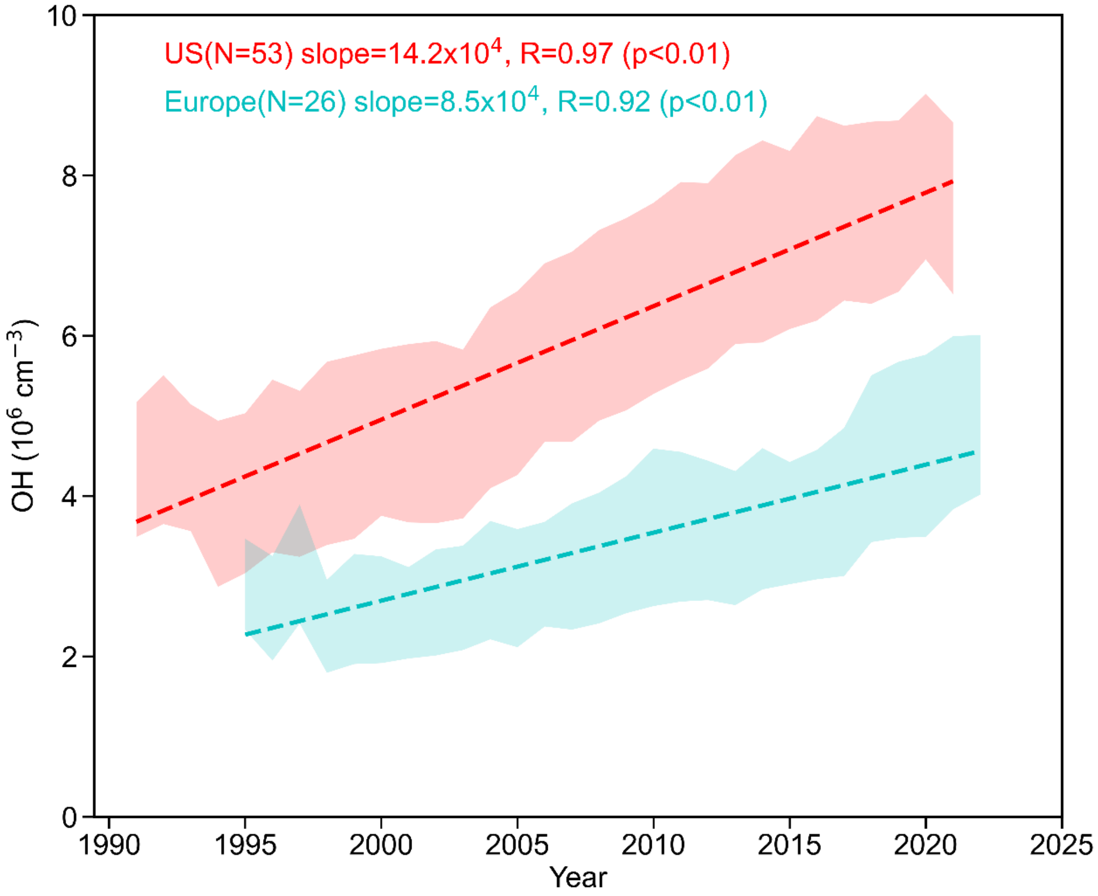

Figure S4. Trends of annual mean OH in the United States (N = 53) and Europe (N = 26) over 1991–2021 and 1995–2022, respectively. Shaded areas represent the range of mean values ± 0.5 standard deviation for each region.

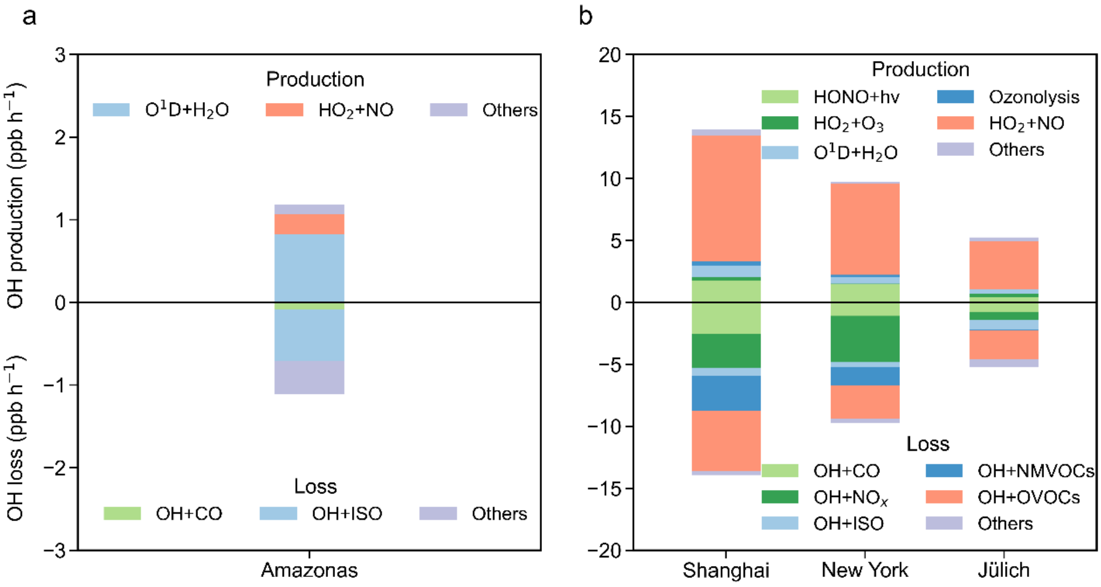

Figure S5. OH budgets between pristine (a) and present-day (b) scenarios. Noontime (11:00–13:00 LT) production and loss rates of OH in Amazonas[4], Shanghai, New York and Jülich, respectively.

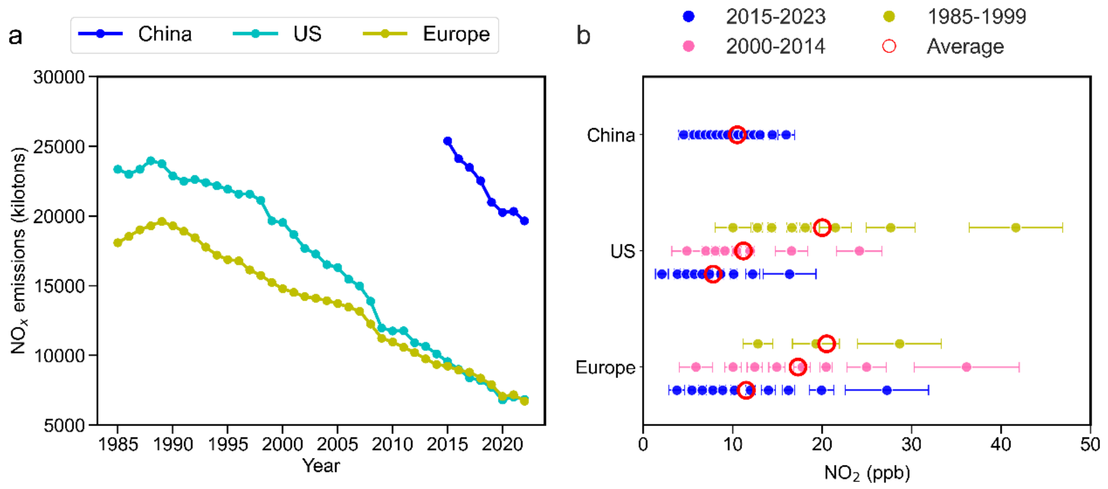

Figure S6. Annual trends of anthropogenic nitrogen dioxide emissions (a) and daytime NO_2 concentrations (b) in China, the United States and Europe over 1985–1999 (N=94, 27), 2000–2014 (N=102, 101), 2015–2023 (N=331, 140, 244), respectively.

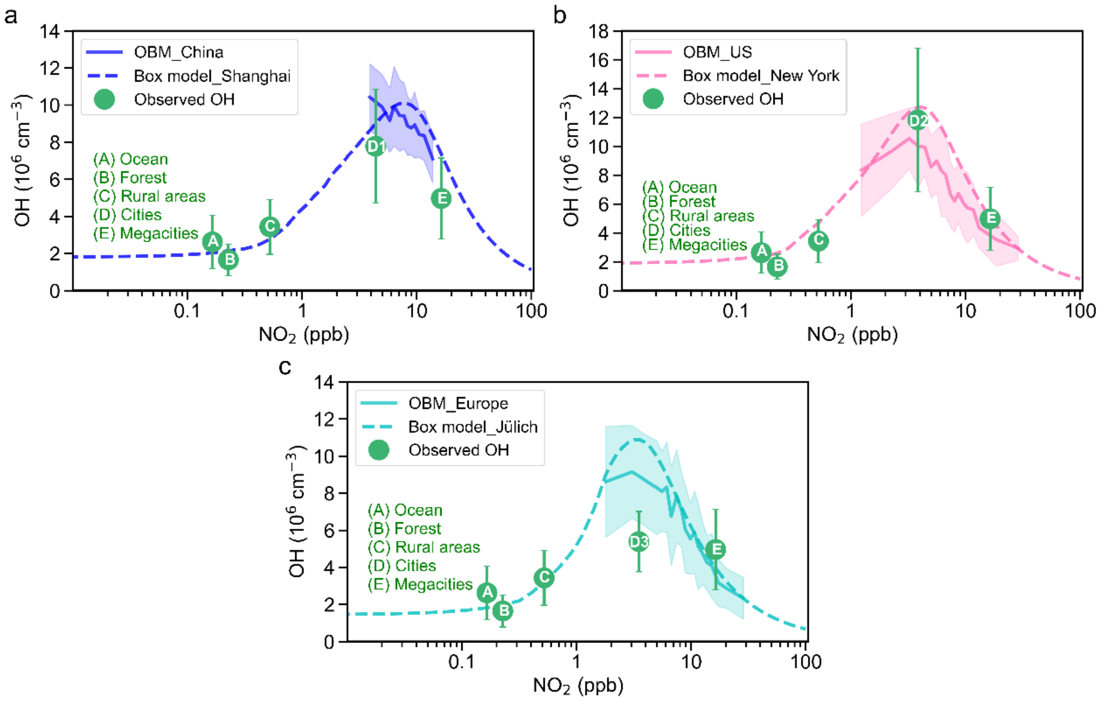

Figure S7. The dependence of OH on NO_2. Noontime (11:00–13:00 LT) OH concentrations derived by the OBM for China (September 2015–2023; N = 331) (a, blue curve with 1-sigma blue shade), the United States (July 1985–2023; N = 535) (b, pink curve with 1-sigma pink shade) and Europe (August 1985–2023; N = 594) (c, cyan curve with 1-sigma cyan shade), are compared to simulations by the chemical box model for China (Shanghai, blue dashed line), the United States (New York, pink dashed line), Germany (Jülich, cyan dashed line), respectively, and observations (green dots with one standard deviation vertical bars). Points D1, D2, D3 represent measurements in Chinese cities, US cities and European cities, respectively. Shaded areas represent the range of mean values ±1 standard deviation for each region.

References

1. Song K, Liu R, Wang Y *et al.* Observation-based analysis of ozone production sensitivity for two persistent ozone episodes in Guangdong, China. *Atmos Chem Phys* 2022; **22**(12): 8403-8416.
2. Rohrer F, Lu K, Hofzumahaus A *et al.* Maximum efficiency in the hydroxyl-radical-based self-cleansing of the troposphere. *Nat Geosci* 2014; **7**(8): 559-563.
3. Cho C, Fuchs H, Hofzumahaus A *et al.* Experimental chemical budgets of OH, HO_2, and RO_2 radicals in rural air in western Germany during the JULIAC campaign 2019. *Atmos Chem Phys* 2023; **23**(3): 2003-2033.
4. Jeong D, Seco R, Emmons L *et al.* Reconciling Observed and Predicted Tropical Rainforest OH Concentrations. *J Geophys Res Atmos* 2022; **127**(1): e2020JD032901.
5. Whalley LK, Edwards PM, Furneaux KL *et al.* Quantifying the magnitude of a missing hydroxyl radical source in a tropical rainforest. *Atmos Chem Phys* 2011; **11**(14): 7223-7233.
6. Mao J, Ren X, Zhang L *et al.* Insights into hydroxyl measurements and atmospheric oxidation in a California forest. *Atmos Chem Phys* 2012; **12**(17): 8009-8020.
7. Griffith SM, Hansen RF, Dusanter S *et al.* OH and HO_2 radical chemistry during PROPHET 2008 and CABINEX 2009 – Part 1: Measurements and model comparison. *Atmos Chem Phys* 2013; **13**(11): 5403-5423.
8. Bottorff B, Lew MM, Woo Y *et al.* OH, HO_2, and RO_2 radical chemistry in a rural forest environment: measurements, model comparisons, and evidence of a missing radical sink. *Atmos Chem Phys* 2023; **23**(18): 10287-10311.
9. Feiner PA, Brune WH, Miller DO *et al.* Testing atmospheric oxidation in an Alabama forest. *J Atmos Sci* 2016; **73**(12): 4699-4710.
10. Tan D, Faloona I, Simpas J *et al.* HO_x budgets in a deciduous forest: Results from the PROPHET summer 1998 campaign. *J Geophys Res Atmos* 2001; **106**(D20): 24407-24427.
11. Bloss W, Lee J, Heard D *et al.* Observations of OH and HO_2 radicals in coastal Antarctica. *Atmos Chem Phys* 2007; **7**(16): 4171-4185.
12. Hosaynali Beygi Z, Fischer H, Harder HD *et al.* Oxidation photochemistry in the Southern Atlantic boundary layer: unexpected deviations of photochemical steady state. *Atmos Chem Phys* 2011; **11**(16): 8497-8513.
13. Whalley LK, Furneaux KL, Goddard A *et al.* The chemistry of OH and HO_2 radicals in the boundary layer over the tropical Atlantic Ocean. *Atmos Chem Phys* 2010; **10**(4): 1555-1576.
14. Vaughan S, Ingham T, Whalley LK *et al.* Seasonal observations of OH and HO_2 in the remote tropical marine boundary layer. *Atmos Chem Phys* 2012; **12**(4): 2149-2172.
15. Creasey D, Heard D,Lee J. Eastern Atlantic Spring Experiment 1997 (EASE97) 1. Measurements of OH and HO_2 concentrations at Mace Head, Ireland. *J Geophys Res Atmos* 2002; **107**(D10): ACH-3.

16. Ren X, Olson JR, Crawford JH *et al.* HO_x chemistry during INTEX-A 2004: Observation, model calculation, and comparison with previous studies. *J Geophys Res Atmos* 2008; **113**(D5).
17. Carslaw N, Creasey DJ, Heard DE *et al.* Modeling OH, HO_2, and RO_2 radicals in the marine boundary layer: 1. Model construction and comparison with field measurements. *J Geophys Res Atmos* 1999; **104**(D23): 30241-30255.
18. Kanaya Y, Cao R, Akimoto H *et al.* Urban photochemistry in central Tokyo: 1. Observed and modeled OH and HO_2 radical concentrations during the winter and summer of 2004. *J Geophys Res Atmos* 2007; **112**(D21).
19. Kanaya Y, Matsumoto J, Kato S *et al.* Behavior of OH and HO_2 radicals during the Observations at a Remote Island of Okinawa (ORION99) field campaign: 2. Comparison between observations and calculations. *J Geophys Res Atmos* 2001; **106**(D20): 24209-24223.
20. Kanaya Y,Akimoto H. Direct measurements of HO_x radicals in the marine boundary layer: Testing the current tropospheric chemistry mechanism. *Chem Rec* 2002; **2**(3): 199-211.
21. Stevens P, Mather J, Brune WH *et al.* HO_2/OH and RO_2/HO_2 ratios during the Tropospheric OH Photochemistry Experiment: Measurement and theory. *J Geophys Res Atmos* 1997; **102**(D5): 6379-6391.
22. Mallik C, Tomsche L, Bourtsoukidis E *et al.* Oxidation processes in the eastern Mediterranean atmosphere: evidence from the modelling of HO_x measurements over Cyprus. *Atmos Chem Phys* 2018; **18**(14): 10825-10847.
23. Eisele FL, Mount GH, Tanner D *et al.* Understanding the production and interconversion of the hydroxyl radical during the Tropospheric OH Photochemistry Experiment. *J Geophys Res Atmos* 1997; **102**(D5): 6457-6465.
24. Eisele FL, Mount GH, Fehsenfeld FC *et al.* Intercomparison of tropospheric OH and ancillary trace gas measurements at Fritz Peak Observatory, Colorado. *J Geophys Res Atmos* 1994; **99**(D9): 18605-18626.
25. Berresheim H, Plass-Dülmer C, Elste T *et al.* OH in the coastal boundary layer of Crete during MINOS: Measurements and relationship with ozone photolysis. *Atmos Chem Phys* 2003; **3**(3): 639-649.
26. Price P, Bottorff B, Jenkins J *et al.* Re-assessing hydroxyl radical chemistry in the atmosphere: Instrument interferences may explain previous measurement discrepancies. *Commun Earth Environ* 2025; **6**(1): 325.
27. Sinha V, Williams J, Diesch JM *et al.* Constraints on instantaneous ozone production rates and regimes during DOMINO derived using in-situ OH reactivity measurements. *Atmos Chem Phys* 2012; **12**(15): 7269-7283.
28. Woodward-Massey R, Sommariva R, Whalley LK *et al.* Radical chemistry at a UK coastal receptor site–Part 2: experimental radical budgets and ozone production. *Atmos Chem Phys* 2022; **2022**: 1-37.
29. Holland F, Hofzumahaus A, Schäfer J *et al.* Measurements of OH and HO_2 radical concentrations and photolysis frequencies during BERLIOZ. *J Geophys Res Atmos* 2003; **108**(D4): 8246.
30. Hofzumahaus A, Rohrer F, Lu K *et al.* Amplified trace gas removal in the

troposphere. *Science* 2009; **324**(5935): 1702-1704.

31. Lu K, Rohrer F, Holland F *et al.* Observation and modelling of OH and HO_2 concentrations in the Pearl River Delta 2006: a missing OH source in a VOC rich atmosphere. *Atmos Chem Phys* 2012; **12**(3): 1541-1569.
32. Mao J, Ren X, Chen S *et al.* Atmospheric oxidation capacity in the summer of Houston 2006: Comparison with summer measurements in other metropolitan studies. *Atmos Environ* 2010; **44**(33): 4107-4115.
33. Zou Z, Chen Q, Xia M *et al.* OH measurements in the coastal atmosphere of South China: possible missing OH sinks in aged air masses. *Atmos Chem Phys* 2023; **23**(12): 7057-7074.
34. Woodward-Massey R, Sommariva R, Whalley LK *et al.* Radical chemistry and ozone production at a UK coastal receptor site. *Atmos Chem Phys* 2023; **23**(22): 14393-14424.
35. Forberich O, Pfeiffer T, Spiekermann M *et al.* Measurement of the Diurnal Variation of the OH Radical Concentration and Analysis of the Data by Modelling. *J Atmos Chem* 1999; **33**: 155-181.
36. Lu KD, Hofzumahaus A, Holland F *et al.* Missing OH source in a suburban environment near Beijing: observed and modelled OH and HO_2 concentrations in summer 2006. *Atmos Chem Phys* 2013; **13**(2): 1057-1080.
37. Michoud V, Kukui A, Camredon M *et al.* Radical budget analysis in a suburban European site during the MEGAPOLI summer field campaign. *Atmos Chem Phys* 2012; **12**(24): 11951-11974.
38. Tan Z, Fuchs H, Lu K *et al.* Radical chemistry at a rural site (Wangdu) in the North China Plain: observation and model calculations of OH, HO_2 and RO_2 radicals. *Atmos Chem Phys* 2017; **17**(1): 663-690.
39. Ma X, Tan Z, Lu K *et al.* OH and HO_2 radical chemistry at a suburban site during the EXPLORE-YRD campaign in 2018. *Atmos Chem Phys* 2022; **22**(10): 7005-7028.
40. Ren X, van Duin D, Cazorla M *et al.* Atmospheric oxidation chemistry and ozone production: Results from SHARP 2009 in Houston, Texas. *J Geophys Res Atmos* 2013; **118**(11): 5770-5780.
41. Kanaya Y, Hofzumahaus A, Dorn HP *et al.* Comparisons of observed and modeled OH and HO_2 concentrations during the ambient measurement period of the HOxComp field campaign. *Atmos Chem Phys* 2012; **12**(5): 2567-2585.
42. Yang X, Lu K, Ma X *et al.* Observations and modeling of OH and HO_2 radicals in Chengdu, China in summer 2019. *Sci Total Environ* 2021; **772**: 144829.
43. Zhang G, Hu R, Xie P *et al.* Intensive photochemical oxidation in the marine atmosphere: evidence from direct radical measurements. *Atmos Chem Phys* 2024; **24**(3): 1825-1839.
44. Whalley LK, Stone D, Dunmore R *et al.* Understanding in situ ozone production in the summertime through radical observations and modelling studies during the Clean air for London project (ClearfLo). *Atmos Chem Phys* 2018; **18**(4): 2547-2571.
45. Tan Z, Rohrer F, Lu K *et al.* Wintertime photochemistry in Beijing: observations

of RO_x radical concentrations in the North China Plain during the BEST-ONE campaign. *Atmos Chem Phys* 2018; **18**(16): 12391-12411.

46. Yang X, Lu K, Ma X *et al.* Radical chemistry in the Pearl River Delta: observations and modeling of OH and HO_2 radicals in Shenzhen in 2018. *Atmos Chem Phys* 2022; **22**(18): 12525-12542.
47. Griffith SM, Hansen RF, Dusanter S *et al.* Measurements of hydroxyl and hydroperoxy radicals during CalNex-LA: Model comparisons and radical budgets. *J Geophys Res Atmos* 2016; **121**(8): 4211-4232.
48. Tan Z, Ma X, Lu K *et al.* Direct evidence of local photochemical production driven ozone episode in Beijing: A case study. *Sci Total Environ* 2021; **800**: 148868.
49. Tan Z, Lu K, Hofzumahaus A *et al.* Experimental budgets of OH, HO_2, and RO_2 radicals and implications for ozone formation in the Pearl River Delta in China 2014. *Atmos Chem Phys* 2019; **19**(10): 7129-7150.
50. Slater EJ, Whalley LK, Woodward-Massey R *et al.* Elevated levels of OH observed in haze events during wintertime in central Beijing. *Atmos Chem Phys* 2020; **20**(23): 14847-14871.
51. Whalley LK, Slater EJ, Woodward-Massey R *et al.* Evaluating the sensitivity of radical chemistry and ozone formation to ambient VOCs and NO_x in Beijing. *Atmos Chem Phys* 2021; **21**(3): 2125-2147.
52. Zhang G, Hu R, Xie P *et al.* Observation and simulation of HO_x radicals in an urban area in Shanghai, China. *Sci Total Environ* 2022; **810**: 152275.
53. Ma X, Tan Z, Lu K *et al.* Winter photochemistry in Beijing: Observation and model simulation of OH and HO_2 radicals at an urban site. *Sci Total Environ* 2019; **685**: 85-95.
54. George LA, Hard TM,O'Brien RJ. Measurement of free radicals OH and HO_2 in Los Angeles smog. *J Geophys Res Atmos* 1999; **104**(D9): 11643-11655.
55. Dusanter S, Vimal D, Stevens P *et al.* Measurements of OH and HO_2 concentrations during the MCMA-2006 field campaign–Part 2: Model comparison and radical budget. *Atmos Chem Phys* 2009; **9**(18): 6655-6675.
